# Meaningful ciphertext image encryption via ND-ICNTC chaos and histogram reorganization with steganalysis-based evaluation

**DOI:** 10.1016/j.isci.2026.116571

**Published:** 2026-07-02

**Authors:** Shiwei Jing, Jianjun Li, Yizhen Huang

**Affiliations:** 1 School of Compute Science and Technology, Hangzhou Dianzi University, Hangzhou 310018, China; 2 School of Information Science and Technology, Hangzhou Normal University, Hangzhou 310001, China; 3 Information Engineering College, Jinhua University of Vocational Technology, University Zone, Jinhua 321017, China

**Keywords:** Image Encryption, Meaningful Ciphertext, ND-ICNTC chaotic, Histogram Reorganization, Steganalysis

## Abstract

To address the common limitations of existing visually meaningful ciphertext image encryption schemes, including low ciphertext quality and limited steganalysis resistance, this paper proposes a visually meaningful ciphertext image encryption algorithm based on an *N*-dimensional infinite-collapse non-adjacent three-term coupling (ND-ICNTC) chaotic system and a histogram-reorganizing strategy. The plaintext image is converted into a noise-like ciphertext via compression, histogram reconstruction, and bit-level scrambling and then embedded into a cover image. The histogram reconstruction and scrambling significantly expand the pixel-value distribution, enhancing distinguishability from conventional ciphertexts and improving data security, while also providing a more favorable embedding distribution that enhances visual quality. The ND-ICNTC system features large Lyapunov exponents, wide chaotic parameter range, uniformly distributed attractors, and strong randomness, while its non-adjacent three-term coupling balances diffusion rate and computational cost. Comprehensive security analyses, including cryptographic evaluation and statistical/learning-based steganalysis, confirm high security, superior visual quality, and strong undetectability.

## Highlights

• A meaningful image encryption scheme is proposed, offering good ciphertext quality
• Steganalysis verifies ciphertext imperceptibility, ensuring secure transmission
• ND-ICNTC balances complexity and diffusion speed, with strong chaos performance

## Graphical abstract

**Figure:**
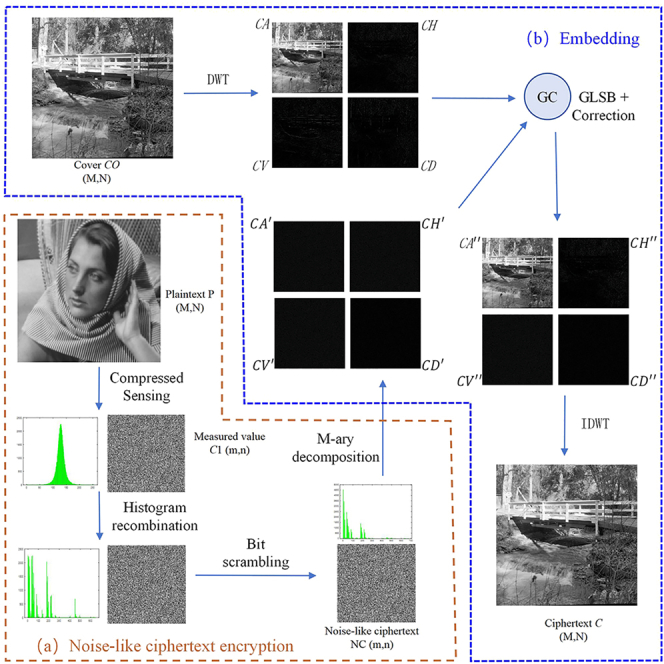

## Teaser

Applied sciences; Data encryption

## Introduction

### *Background*

With the continuous advancement of society and technology, the acquisition, transmission, and storage of information have become increasingly convenient, frequently involving sensitive data such as personal privacy and military secrets. Owing to their rich expressive capability and intuitive representation, images are extensively utilized in modern information systems, rendering image security a critical research issue. To address this, researchers have proposed numerous image encryption algorithms, such as pixel-value scrambling and diffusion schemes,^1^^,^^2^ bit-level scrambling methods,^3^ filtering diffusion,^4^ DNA-based coding techniques,^5^^,^^6^ compressive sensing-based encryption,^7^ optical encryption approaches,^8^^,^^9^ quantum encryption frameworks,^10^ and deep neural network-based encryption methods.^11^ These algorithms encrypt plaintext into noise-like images for transmission to the user, ensuring data security.

However, ciphertext from traditional image encryption algorithms is noise-like, rendering it easily detectable during transmission. To address this, Bao et al.^12^ proposed a visually meaningful image encryption scheme in 2015 that embeds noise-like ciphertext into the two high-frequency wavelet subbands of a cover image by replacing coefficients with the ciphertext’s hundreds/tens and units digits, respectively. Yet, direct substitution introduces noticeable texture artifacts in the cover image, making it susceptible to detection and attack. To alleviate this issue, several improved embedding strategies have been proposed to enhance ciphertext quality.^13^ For instance, Yang et al.^14^ replaced direct substitution with additive embedding between ciphertext digits and high-frequency coefficients of the cover image. Similarly, Ye et al.^15^ embedded the hundreds, tens, and units digits of the ciphertext into the units digit of the cover’s high-frequency coefficients. While these methods preserve more high-frequency information and improve visual quality, decimal-based decomposition allows the summed values to reach up to 20, which is suboptimal for minimizing cover distortion. Further improvements were achieved in Yang et al. and Wu et al.^16^^,^^17^ through binary decomposition of the ciphertext, where 3, 3, and 2 bits are embedded into different high-frequency subbands, respectively. This strategy reduces the maximum possible sum after decomposition to 17, thereby further improving ciphertext quality. Subsequently, Yu et al.^18^ extended the embedding process to low-frequency subbands, allocating 2 bits to each of four frequency bands to further reduce cover modification. Yang et al. and Ye et al.^19^^,^^20^ introduced a 2^*k*^ correction mechanism and proposed a weighted distribution of the 8-bit ciphertext among frequency bands (1, 2, 2, and 3 bits from low to high frequency), which significantly enhanced ciphertext quality. Yang et al.^21^ further optimized the bit allocation per frequency using Mary decomposition. While most of the aforementioned methods perform embedding in the transform domain, spatial-domain approaches have also been investigated, including least significant bit (LSB) replacement^22^^,^^23^ and matrix coding techniques.^24^^,^^25^ To further enhance visual quality, the 2^*k*^ correction mechanism has also been introduced into spatial-domain LSB embedding.^26^^,^^27^ Recently, deep-learning-based embedding has also been proposed to enhance transmission security.^28^ Both spatial-domain and transform-domain embedding suffer from a severe decoupling from the preceding encryption process: encryption is not designed to accommodate embedding, nor is embedding tailored to encryption, limiting further gains in ciphertext quality.

Owing to their strong sensitivity to initial conditions and system parameters, as well as their inherent pseudorandomness, chaotic systems are highly compatible with the requirements of cryptography.^29^ Moreover, employing chaotic sequences as encryption control parameters can effectively enlarge the key space, simplify key distribution and management, and significantly enhance key sensitivity.^30^ Consequently, a wide range of image encryption algorithms based on chaotic systems has been proposed. One-dimensional discrete chaotic systems are characterized by simple structures and fast sequence generation.^31^^,^^32^ However, they often suffer from weak chaotic behavior, narrow chaotic parameter ranges, limited parameter variability, and simple trajectories, making their initial conditions and dynamical evolution relatively easy to predict.^33^ In contrast, high-dimensional continuous chaotic systems exhibit stronger chaotic properties and more complex dynamical behavior but usually require longer sequence generation times and higher computational cost.^33^^,^^34^ To improve chaotic performance while maintaining efficiency, many studies have constructed higher dimensional chaotic models by cascading or coupling one-dimensional systems. For instance, Gao et al.^35^ entangled the logistic map with the Rulkov neuron to obtain a two-dimensional chaotic system with stronger chaotic properties. Zheng et al.^36^ combined the sine map, Gaussian map, and iterative map to develop a two-dimensional discrete hyperchaotic system termed the Infinite Gaussian Sine Chaotic Map. Liao et al.^37^ coupled logistic and sine maps to construct a discrete three-dimensional hyperchaotic system. Lin et al.^38^ introduced a nonlinear controller to achieve a three-dimensional chaotic system whose complexity increases logarithmically with the control parameter. Although these systems outperform one-dimensional chaotic maps, their fixed dimensionality limits flexibility, and their low-dimensional structures restrict parallel processing capability.

**Figure 1:**
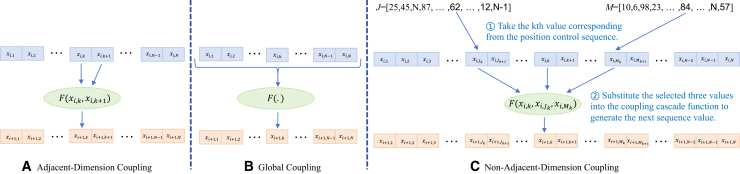
Three coupling schemes of *N*-dimensional chaotic systems (A) Adjacent-Dimension Coupling scheme. (B) Global Coupling scheme. (C) Non-Adjacent-Dimension Coupling scheme.

To overcome these limitations, several *N*-dimensional discrete chaotic systems have been proposed using cascading strategies. For instance, sine-based entanglement between adjacent dimensions was employed in Jing et al.^39^ to construct an *N*-dimensional chaotic system. Similarly, Li et al.^40^ introduced a parameterized coupling term preceding a sine chaotic map to extend the system dimensionality, while Wang et al.^41^ adopted a related approach with a more complex sine-based coupling structure. In these systems, each dimension is coupled with another, as illustrated in Figure 1A, preserving structural simplicity and computational efficiency.^39^ However, cross-dimensional diffusion remains relatively slow, as a perturbation introduced in a single dimension typically requires approximately *N* iterations to propagate throughout the entire system.^42^ Beyond pairwise coupling schemes, another class of approaches achieves global coupling among all dimensions, as illustrated in Figure 1B. For example, Wang et al. and Liu et al.^43^^,^^44^ employ *N*-dimensional polynomial functions to fuse information from all dimensions, followed by modular operations to constrain the outputs within a predefined range. Alternatively, Cao et al. and Zhao et al.^45^^,^^46^ propose a simpler strategy in which each dimension is first processed by a one-dimensional chaotic map, and the resulting outputs are aggregated through summation and bounded using trigonometric functions. These fully coupled *N*-dimensional chaotic systems enable rapid diffusion, as all dimensions interact simultaneously. However, this advantage comes at the expense of increased structural complexity and rapidly growing computational cost as the system dimensionality increases.

### *Motivation*

Analysis of visually meaningful ciphertext encryption and *N*-dimensional chaos reveals the following issues.

• For most visually meaningful ciphertext schemes, encryption and embedding are entirely decoupled. Image encryption and steganographic embedding are treated as separate tasks, leaving ciphertext quality dependent solely on the steganography algorithm—fundamentally limiting performance. Yet, visually meaningful ciphertext embedding differs fundamentally from traditional steganography, as the embedded values are generated by a carefully designed encryption algorithm rather than being random sequences. This distinction creates an opportunity to guide encryption toward generating embedding-friendly values, thereby improving ciphertext quality.
• For *N*-dimensional chaotic systems, both adjacent coupling and global coupling schemes exhibit distinct advantages, yet each also suffers from notable limitations. In practice, these two coupling strategies present mutually exclusive characteristics in terms of computational efficiency and diffusion capability. An effective coupling structure should simultaneously achieve high computational efficiency and rapid cross-dimensional diffusion. Inspired by the non-adjacent coupling strategy proposed for coupled map lattice spatiotemporal chaos,^42^ where coupling among only three non-adjacent lattices is illustrated in Figure 1C, this work extends the concept to *N*-dimensional chaotic systems to obtain a structure that is both simple and capable of rapid diffusion.

Based on these observations, a novel visually meaningful ciphertext image encryption algorithm is proposed in this paper, which offers the following three main advantages.

• A histogram reorganization strategy is introduced in the noise-like ciphertext generation stage. This strategy enables the ciphertext distribution to break through the conventional pixel value range of [0, 255], thereby increasing confusion and enhancing resistance against statistical analysis attacks. More importantly, histogram reorganization provides an optimal distribution tailored to the subsequent embedding process. By aligning the ciphertext histogram with the optimal distribution under given embedding constraints, the proposed strategy effectively minimizes the visual distortion of the cover image and significantly improves the visual quality of the meaningful ciphertext.
• A novel *N*-dimensional infinite-collapse non-adjacent three-term coupling (ND-ICNTC) chaotic system is designed based on a non-adjacent three-term coupling structure. This design not only ensures efficient pseudorandom sequence generation but also reduces the required number of diffusion iterations from *N* to ⌈ log_2_(*N* + 1)⌉ − 1, achieving fast cross-dimensional diffusion with low computational complexity. The complex dynamical properties of the proposed system are verified through attractor analysis, Lyapunov exponent (LE) evaluation, and randomness tests, demonstrating its suitability as a cryptographic pseudorandom number generator.
• Extensive experiments conducted on multiple datasets demonstrate that the proposed encryption algorithm achieves ciphertext peak signal-to-noise ratio (PSNR) values of 51 dB in the spatial domain and 49 dB in the wavelet domain, significantly outperforming existing visually meaningful ciphertext schemes in terms of visual quality. In addition, the proposed method exhibits strong robustness against both conventional statistical steganalysis and deep-learning-based steganalysis models, indicating high imperceptibility and steganographic security, thereby ensuring secure ciphertext transmission.

The remainder of this paper is organized as follows. The ND-ICNTC chaotic section introduces the ND-ICNTC chaotic system and presents its experimental analysis. The noise like ciphertext generation and embedding section explains the principle of histogram shifting, as well as the generation of noise-like ciphertext and its embedding process corresponding to histogram reorganization. The encryption and decryption process section details the encryption and decryption procedures. Experimental results and performance analyses are provided in results. Finally, concluding remarks are given in discussion.

### ND-ICNTC chaotic system

#### Non-adjacent coupling scheme

To balance diffusion speed and computational complexity, this paper introduces a non-adjacent coupling scheme inspired by coupled map lattice spatiotemporal chaos.^42^ As shown in Figure 1C, the control position sequences *J* and *M* can be obtained using the Arnold cat map or by sorting a random sequence and taking the sorted indices. Any sequence that satisfies the following two requirements is applicable for determining the cascading positions:

(1) the sequence contains all integers from 1 to *N*;
(2) the sequence exhibits a random distribution.

In this paper, *N* values generated by the logistic map are employed and subsequently sorted to obtain the corresponding indices. Specifically, a sequence of length *N* is first generated according to Equation 1. The generated sequence is then sorted as described in Equation 2, and the resulting index order is used as the positional sequence. During the subsequent *N*-dimensional chaotic iterations, these positional indices remain fixed and do not require further updating.

(Equation 1) $$x_{i+1}=\mu x_{i}\left(1-x_{i}\right)$$

(Equation 2) K=argsort(X)

This coupling structure allows each dimension to transmit its state information to at most two target dimensions per iteration. After *t* iterations, the maximum number of dimensions to which the information of a single source node can propagate is given by

(Equation 3) $$S\left(t\right)=1+2+2^{2}+\cdots +2^{t}=2^{t+1}-1$$

achieving an exponential growth rate. To propagate information to all *N* dimensions, the following condition must be satisfied:

(Equation 4) $$2^{t+1}-1\geq N\;\Rightarrow \;t\geq \left\lceil \log_{2}\left(N+1\right)\right\rceil -1$$

Taking *N* = 8 as an example, the ideal number of iterations is $\left\lceil \log_{2}\left(8+1\right)\right\rceil -1=3$. Let the random control sequences be *J* = [5, 1, 6, 3, 7, 2, 4, 8] and *M* = [6, 8, 4, 5, 3, 7, 1, 2], with the perturbation factor applied to the first dimension. The perturbation process is as follows.

• Round 1: through *J* and *K*, the perturbation from the first dimension can be transmitted to dimensions 2 and 7. After this round, the perturbed dimensions are {1, 2, 7}.
• Round 2: each of the two newly added dimensions from the previous round propagates to two new dimensions, {6, 8} and {5, 6}, respectively. After removing duplicates, the cumulative perturbed dimensions are {1, 2, 7, 6, 8, 5}.
• Round 3: the three newly added dimensions propagate to new dimensions as follows: {3, 1}, {8, 2}, and {1, 4}. After removing duplicates, the cumulative perturbed dimensions are {1, 2, 7, 6, 8, 5, 3, 4}. The perturbation has now propagated to every dimension.

Thus, despite the presence of duplicate nodes during propagation, the final outcome still achieves the ideal diffusion speed. Nevertheless, duplicate nodes in the iterative process inevitably reduce diffusion efficiency, highlighting the importance of control sequence design. The proximity of diffusion speed to the ideal value across different dimensions is further analyzed in comparative analysis of diffusion rate and iteration time.

#### Infinite collapse mapping and ND-ICNTC

In Figure 1, *F* denotes the cascade coupling function. In this paper, a novel cascade coupling function based on infinite collapse mappings is proposed. The resulting chaotic system is referred to as the ND-ICNTC chaos. The infinite collapse map is defined as^47^

(Equation 5) $$x_{i+1}=sin\left(\mu /x_{i}\right)$$

with *μ* > 0 and *x* ∈ [−1, 0) ∪ (0, 1]. This chaotic system possesses infinitely many unstable fixed points near the origin. Specifically, as *x* → 0, we have *a*/*x* → *∞*, so that sin(*a*/*x*) oscillates infinitely often, yielding infinitely many solutions in any neighborhood of the origin. Consequently, the trajectory jumps continuously among infinitely many unstable fixed points without converging to any periodic orbit, thereby enhancing the complexity and randomness of the generated sequences.^47^ To preserve this high level of randomness, we couple two infinite collapse maps to form an *N*-dimensional chaotic system. Meanwhile, to overcome the non-uniform distribution caused by the sine function, we replace the outermost sine function with a modulo function, resulting in the proposed ND-ICNTC chaotic system, which is defined as follows:

(Equation 6) $$x_{i+1,k}=F\left(x_{i,J_{k}},x_{i,k},x_{i,M_{k}}\right)=\left(\mu 1/\left(x_{i,k}sin\left(\mu 2/\left(x_{i,J_{k}}x_{i,M_{k}}\right)\right)\right)\right)mod1$$

with $x\in \left(\left(0,1\right)\right]$. For *μ*1 > 0 and *μ*_2_ > 0, the system is in a chaotic state.

#### Analysis of fixed points

Since the mappings *J* and *M* are randomly assigned, deriving general analytical results is challenging. To facilitate tractable analysis, we therefore consider the following representative special case:

(Equation 7) $$x_{i+1,k}=\left(\frac{\mu_{1}}{x_{i,k}\sin\;\left(\frac{\mu_{2}}{x_{i,k+1}^{2}}\right)}\right)\;\mathrm{mod}\;1,\;k=1,2,\ldots ,N$$

A fixed point $x_{k}^{*}$ satisfies

(Equation 8) $$x_{k}^{*}=x_{i+1,k}=x_{i,k}$$

Removing the modulo operation, we introduce integers $n_{k}\in Z$ such that

(Equation 9) $$x_{k}^{*}+n_{k}=\frac{\mu_{1}}{x_{k}^{*}\sin\;\left(\frac{\mu_{2}}{{\left(x_{k+1}^{*}\right)}^{2}}\right)}$$

This leads to the following nonlinear algebraic system:

(Equation 10) $$f_{k}\left(x_{k}^{*},x_{k+1}^{*}\right)=x_{k}^{*}\left(x_{k}^{*}+n_{k}\right)\;\sin\;\left(\frac{\mu_{2}}{{\left(x_{k+1}^{*}\right)}^{2}}\right)-\mu_{1}=0$$

As *x*_*k*+1_ → 0, the term $\sin\;\left(\frac{\mu_{2}}{{\left(x_{k+1}\right)}^{2}}\right)$ oscillates infinitely within the interval [−1, 1]. Consequently, $x_{k}\left(x_{k}+n_{k}\right)\sin\;\left(\frac{\mu_{2}}{{\left(x_{k+1}\right)}^{2}}\right)$ exhibits infinitely many oscillations in any neighborhood of zero, implying that the equation $x_{k}\left(x_{k}+n_{k}\right)\sin\;\left(\frac{\mu_{2}}{{\left(x_{k+1}\right)}^{2}}\right)=\mu_{1}$ admits infinitely many solutions in (0, 1). This indicates the existence of infinitely many fixed points.

To investigate stability, we compute the partial derivatives of $G\left(x_{k},x_{k+1}\right)=\frac{\mu_{1}}{x_{k}\sin\left(\frac{\mu_{2}}{x_{k+1}^{2}}\right)}$:

(Equation 11) $$\frac{\partial G}{\partial x_{k}}=-\frac{\mu_{1}}{{\left(x_{k}\right)}^{2}\sin\left(\frac{\mu_{2}}{x_{k+1}^{2}}\right)}$$

(Equation 12) $$\frac{\partial G}{\partial x_{k+1}}=\frac{2\mu_{1}\mu_{2}\cos\left(\frac{\mu_{2}}{x_{k+1}^{2}}\right)}{x_{k}{\left(x_{k+1}\right)}^{3}\;\sin^{2}\left(\frac{\mu_{2}}{x_{k+1}^{2}}\right)}$$

For analytical convenience, we focus on the synchronized fixed point

$$x_{1}^{*}=x_{2}^{*}=\cdots =x_{N}^{*}=x^{*}$$

At this point, the *N* × *N* Jacobian matrix *J* evaluated at the synchronized fixed point takes the form of a circulant-like bidiagonal matrix

(Equation 13) $$J=\left[\begin{array}{ccccc} a & b & 0 & \cdots & 0\\ 0 & a & b & \cdots & 0\\ \vdots & \vdots & \ddots & \ddots & \vdots \\ 0 & 0 & \cdots & a & b\\ b & 0 & \cdots & 0 & a \end{array}\right]$$

where

(Equation 14) $$a=-\frac{\mu_{1}}{{\left(x^{*}\right)}^{2}\sin\left(\frac{\mu_{2}}{{\left(x^{*}\right)}^{2}}\right)}$$

(Equation 15) $$b=\frac{2\mu_{1}\mu_{2}\cos\left(\frac{\mu_{2}}{{\left(x^{*}\right)}^{2}}\right)}{{\left(x^{*}\right)}^{3}\;\sin^{2}\left(\frac{\mu_{2}}{{\left(x^{*}\right)}^{2}}\right)}$$

The eigenvalue spectrum of this specific cyclic bidiagonal matrix is well known and can be obtained from the characteristic polynomial of a cyclic continuation

(Equation 16) $$\det\left(J-\lambda I\right)={\left(a-\lambda \right)}^{N}-{\left(-b\right)}^{N}=0$$

This yields the eigenvalues

(Equation 17) $$\lambda_{j}=a+b\;\cdot \;e^{i\left(\frac{2\pi j}{N}\right)},\;j=0,1,\ldots ,N-1$$

Consequently, the magnitudes of the eigenvalues are

(Equation 18) $$|\lambda_{j}|=\sqrt{a^{2}+b^{2}+2ab\cos\left(\frac{2\pi j}{N}\right)}$$

The maximum magnitude is attained when the cosine term equals 1 (i.e., *j* = 0 or constructive interference):

(Equation 19) $$\underset{j}{\max}|\lambda_{j}|=|a+b|$$

As *x*∗ → 0, the term $\frac{1}{{\left(x^{*}\right)}^{3}}$ in *b* dominates, causing |*b*| → *∞*. Consequently, max |*λ*_*j*_|≫ 1, which ensures that any fixed point sufficiently close to the origin is highly unstable. When $\frac{\mu_{2}}{{\left(x^{*}\right)}^{2}}\approx m\pi$ for integer *m*, the denominator sin(…) → 0. In this regime, |*a*| → *∞* and |*b*| → *∞*, again yielding exploding eigenvalues and strong local instability.

Overall, although the system admits infinitely many fixed points, these points are predominantly unstable. This behavior originates from the reciprocal structure of the mapping, the highly oscillatory sine function with inverse arguments, and the cross-dimensional coupling mechanism. Together, these factors ensure strong sensitivity to initial conditions and provide a theoretical basis for the observed chaotic dynamics.

#### Performance analysis

##### Comparative analysis of diffusion rate and iteration time

**Figure 2:**
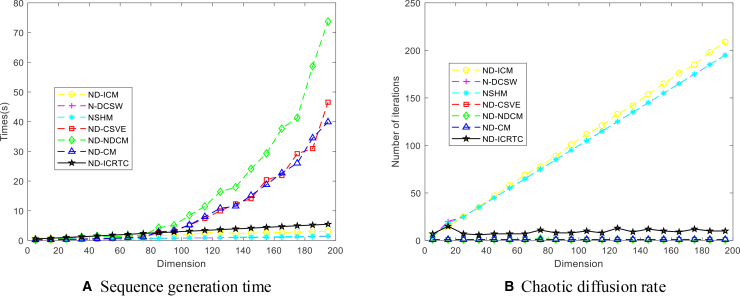
Diffusion rate and iteration time comparison (A) Sequence generation time. (B) Chaotic diffusion rate.

Diffusion describes how a single-bit change in plaintext or seed key leads to significant changes in ciphertext or keystream^42^ and is key to cryptographic security. As sequence generation time directly impacts encryption runtime, a secure algorithm must also be computationally efficient. We therefore compare the diffusion speed and computation time of chaotic systems under the coupling schemes shown in Figure 1 as dimensionality varies. The first category includes N-dimensional infinite collapse discrete chaotic map (ND-ICM),^39^ N-dimensional discrete attractor with sinusoidal waveform (N-DCSW),^40^ and N-dimensional discrete sine hyperchaotic map (NSHM)^41^; the second category includes N-dimensional non-degenerate chaos based on singular value estimation (ND-CSVE),^43^n-dimensional non-degenerate chaotic map (ND-NDCM),^44^ and N-dimensional chaotic map (ND-CM).^45^ Each system is iterated 200,000 times. Diffusion rate is measured by the number of iterations needed for a 0.01 perturbation added to the first dimension’s initial value to propagate through the entire system. Results are shown in Figure 2.

As shown in Figure 2, the first chaotic category exhibits no increase in generation time with dimensionality due to its adjacent coupling mechanism; instead, generation time may even decrease at higher dimensions due to parallelism. For example, ND-ICM requires 10,000 iterations (0.5 s) to generate 200,000 sequences at dimension 20 but only 1,000 iterations (0.1 s) at dimension 200. However, a perturbation requires at least *N* iterations for global diffusion, as each iteration affects only one additional dimension. In contrast, the second category (global coupling) achieves single-iteration diffusion but suffers from sharply increasing computational complexity and generation time with dimensionality. Our proposed scheme increases generation time only marginally with dimensionality—slightly slower than the first category (three dimensions per coupling vs. two) but much faster than the second. Diffusion speed approaches the ideal $\left\lceil \log_{2}\left(N+1\right)\right\rceil -1$, especially at high dimensions—much faster than the first category (*N* iterations) and only slightly slower than the second (single iteration), remaining acceptable. Overall, the proposed *N*-dimensional chaotic system balances diffusion speed and computational complexity, making it suitable as a cryptographic pseudorandom number generator.

##### Analysis of chaotic properties in specific dimensions

**Figure 3:**
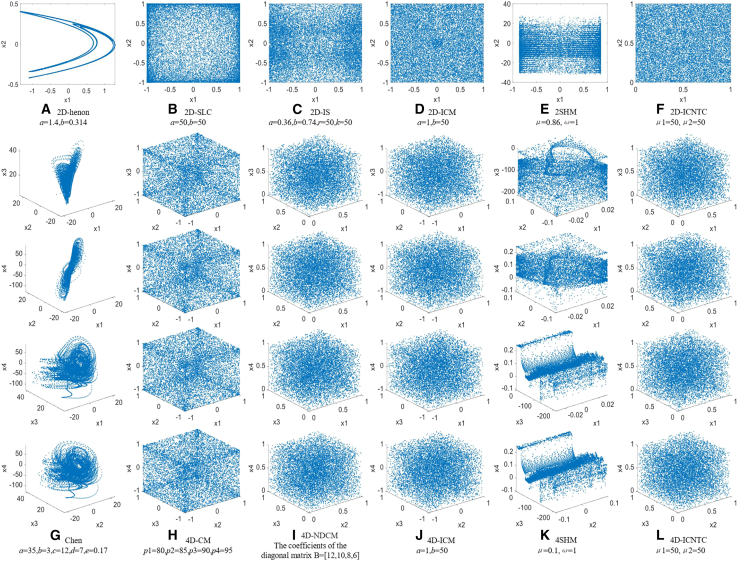
Phase diagram analysis of different chaotic systems (A) 2D-henon: The coefficients *a* = 1.4, *b* = 0.314. (B) 2D-SLC: The coefficients *a* = 50, *b* = 50. (C) 2D-IS: The coefficients *a* = 0.36, *b* = 0.74, *r* = 50, *k* = 50. (D) 2D-ICM: The coefficients *a* = 1, *b* = 50. (E) 2SHM: The coefficients *μ* = 0.86, *ω* = 1. (F) 2D-ICNTC: The coefficients *μ*1 = 50, *μ*2 = 50. (G) Chen: The coefficients *a* = 35, *b* = 3, *c* = 12, *d* = 7, *e* = 0.17. (H) 4D-CM: The coefficients *p*1 = 80, *p*2 = 85, *p*3 = 90, *p*4 = 95. (I) 4D-NDCM: The coefficients of the diagonal matrix B = [12, 10, 8, 6]. (J) 4D-ICM: The coefficients *a* = 1, *b* = 50. (K) 4SHM: The coefficients *μ* = 0.86, *ω* = 1 (L) 4D-ICNTC The coefficients *μ*1 = 50, *μ*2 = 50.

To validate the chaotic properties of ND-ICNTC, this section presents two chaotic maps constructed using the proposed method, namely 2D-ICNTC and 4D-ICNTC chaotic, which are defined by Equations 20 and 21, respectively. For comparison, several representative chaotic systems are considered, including two-dimensional chaotic maps (2D-Henon,^48^ 2D Sin-Linear-Cos [2D-SLC],^25^ and 2D improved sine-trigonometric [2D-IS]^27^), a four-dimensional chaotic system (Chen system^49^), and several *N*-dimensional chaotic systems (ND-ICM,^39^ NSHM,^41^ ND-CM,^45^ and ND-NDCM^44^). For the *N*-dimensional chaotic systems, specific dimensional instances are adopted for evaluation, including 2D-ICM, 4D-ICM, 2SHM, 4SHM, 4D-CM, and 4D-NDCM. The specific dimensional chaotic systems of the *N*-dimensional models are adopted from the original references, with the corresponding system parameters listed in Figure 3.

(Equation 20) $$\left\{\begin{array}{c} x_{i+1,1}=\mu 1/\left(x_{i,1}sin\left(\mu 2/x_{i,2}^{2}\right)\right)mod1\\ x_{i+1,2}=\mu 1/\left(x_{i,2}sin\left(\mu 2/x_{i,1}^{2}\right)\right)mod1 \end{array}\right)$$

(Equation 21) $$\left\{\begin{array}{c} x_{i+1,1}=\mu 1/\left(x_{i,1}sin\left(\mu 2/x_{i,4}x_{i,2}\right)\right)mod1\\ x_{i+1,2}=\mu 1/\left(x_{i,2}sin\left(\mu 2/x_{i,1}x_{i,3}\right)\right)mod1\\ x_{i+1,3}=\mu 1/\left(x_{i,3}sin\left(\mu 2/x_{i,2}x_{i,4}\right)\right)mod1\\ x_{i+1,4}=\mu 1/\left(x_{i,4}sin\left(\mu 2/x_{i,3}x_{i,1}\right)\right)mod1 \end{array}\right)$$

###### Attractor diagram analysis

Figure 3 presents the phase diagrams of various chaotic systems. It can be observed that the phase-space distributions of the 2D-Hénon, 2D-SLC, 2D-IS, 2SHM, Chen, and 4SHM systems exhibit pronounced clustering behavior. In particular, the classical 2D-Henon and Chen systems show clearly distinguishable trajectory patterns, indicating limited ergodicity and weak unpredictability. By contrast, the proposed 2D-ICNTC and 4D-ICNTC chaotic systems demonstrate highly uniform phase-space distributions, implying that the generated sequences possess superior randomness and unpredictability.

###### Bifurcation analysis

**Figure 4:**
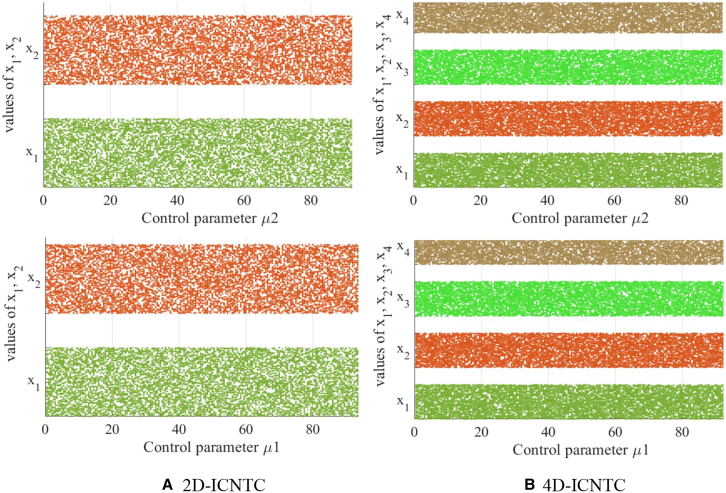
Bifurcation diagrams for different dimensions In the first row, *μ*1 ∈ (0, 100] and *μ*2 = 50; in the second row, *μ*2 ∈ (0, 100] and *μ*1 = 50. (A) 2D-ICNTC bifurcation diagrams. (B) 4D-ICNTC bifurcation diagrams.

A bifurcation diagram is a tool for examining how the complex behavior of a chaotic system changes with control parameters, revealing whether periodic windows or regions with weak dynamics exist. We analyzed the bifurcation diagrams of each dimension for the proposed 2D-ICNTC and 4D-ICNTC systems with $\mu 1\in \left(\left(0,100\right)\right]$ and *μ*2 ∈ (0, 100], as shown in Figure 4. Across a wide range of parameter values, the bifurcation diagrams of all dimensions exhibit densely distributed discrete points with no periodic windows. This indicates that the system is highly complex and that the generated sequences possess strong randomness.

###### LE

**Figure 5:**
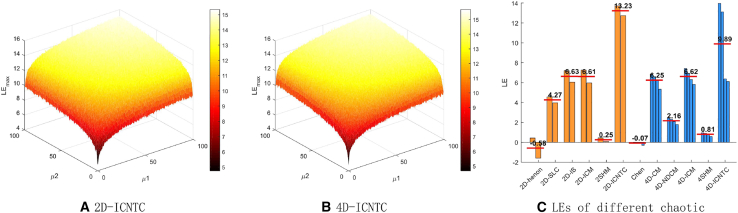
Lyapunov exponent analysis of different chaotic systems (A) 2D-ICNTC. (B) 4D-ICNTC. (C) LEs of diffent chaotic.

The LE is a fundamental metric for characterizing the dynamical behavior of nonlinear systems and for distinguishing periodic motion from chaos; larger LE values correspond to stronger chaotic characteristics. Figures 5A and 5B show the variations of the maximum LE for the 2D-ICNTC and 4D-ICNTC chaotic systems with respect to their parameters, respectively, while Figure 5C illustrates the LE values of different chaotic systems under the parameter settings specified in Figure 3. It can be observed that the LE of ND-ICNTC chaotic system increases as the system parameter grows and remains positive across the entire parameter range, indicating that the system stays in a chaotic regime throughout this domain and that its LE can be tuned via system parameters. Moreover, a comparison of the LE values in Figure 5C reveals that ND-ICNTC chaotic system yields larger LEs than the other chaotic systems, thereby confirming its superior chaotic performance.

###### Sequence randomness

**Figure 6:**
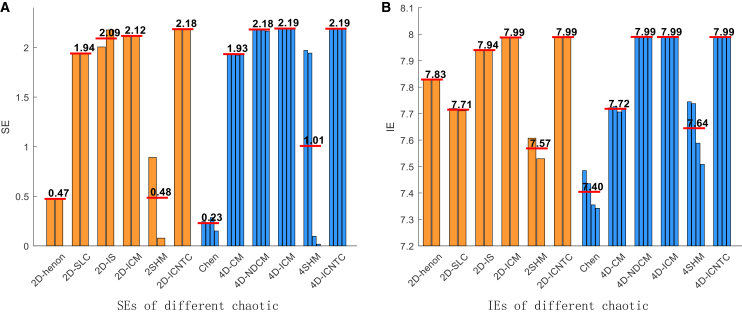
Entropy analysis of different chaotic systems (A) SEs of diffent chaotic. (B) IEs of diffent chaotic.

This paper evaluates the randomness of chaotic sequences using sample entropy (SE) and information entropy (IE). SE quantifies the complexity of a time series by measuring the probability of generating new patterns within the signal. In general, higher entropy values indicate greater sequence complexity, uniformity, and randomness. Since different chaotic systems exhibit distinct value ranges, all sequences are normalized to the integer interval [0,255] when calculating IE, for which the theoretical optimum is 8. Figure 6 illustrates the entropy values of each dimension for all chaotic sequences after 20,000 iterations under the parameter settings shown in Figure 3. It can be observed that both 2D-ICNTC and 4D-ICNTC chaotic exhibit nearly identical and consistently high entropy values across all dimensions, indicating that the chaotic sequences generated by ND-ICNTC chaotic system maintain strong and stable randomness regardless of dimensionality. Furthermore, comparisons with other chaotic models reveal that the entropy values achieved by the proposed ND-ICNTC chaotic system approach the theoretical optimum, providing further evidence of the robust randomness of the generated chaotic sequences.

**Figure 7:**
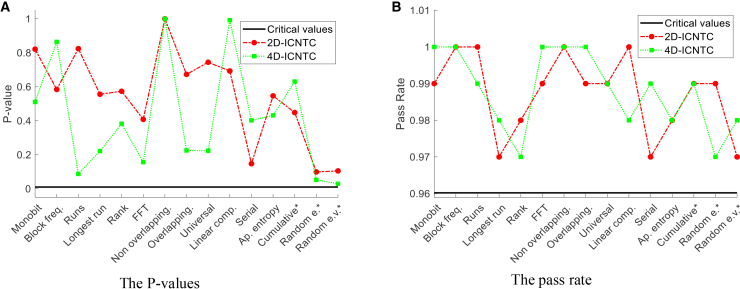
NIST SP800-22 standard test (A) The *P*-values. (B) The pass rate.

To further verify the randomness of the chaotic sequences generated in this paper, the NIST SP800-22 published by the National Institute of Standards and Technology (NIST) is adopted for statistical evaluation. The test suite consists of 15 independent subtests. At a significance level of *α* = 0.01, if the *p* value of a subtest is greater than 0.01, the corresponding sequence is considered to have passed that subtest. Sequences of length 10^8^ are generated using the 2D-ICNTC and 4D-ICNTC, respectively, and are converted into binary sequences with a threshold of 0.5. Each binary sequence is then divided into 100 groups, each of length 10^6^ bits. According to the confidence interval formula $\hat{p}\pm 3\sqrt{\hat{p}\left(1-\hat{p}\right)/m}$ recommended by the NIST SP800-22, where $\hat{p}=1-\alpha =0.99$ and *m* = 100 (the number of groups), the resulting confidence interval is [0.9602, 1.0198]. If the proportion of sequences passing a subtest falls within this interval, statistical evidence supports the randomness of the sequences. Figure 7 presents the test results for both chaotic systems. It can be observed that all *p* values are greater than 0.01, and the passing rates for all subtests fluctuate within the interval [0.9602, 1.0198]. The results demonstrate that the generated chaotic binary sequences successfully pass all NIST subtests, indicating strong randomness and suitability for use as pseudorandom number generators in practical applications.

### Noise-like ciphertext generation and embedding

**Figure 8:**
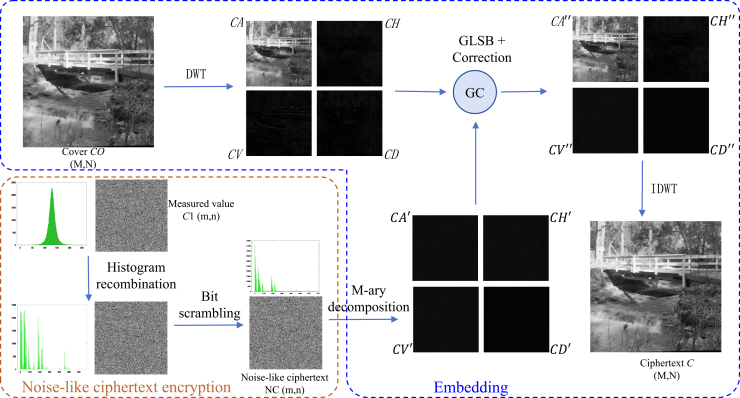
Noise-like encryption and embedding process The embedding process illustrated here is performed in the wavelet transform domain. For spatial-domain embedding, the cover image can be directly and uniformly divided into four sub-blocks.

This section discusses the noise-like ciphertext encryption and embedding process based on the previously generated Gaussian-like distribution ciphertext. First, histogram reorganization and M-ary decomposition-based bit permutation are applied to the Gaussian-like distribution ciphertext to obtain noise-like ciphertext. Subsequently, generalized least significant bit (GLSB) embedding and correction are performed to produce the final ciphertext. The overall procedure is illustrated in Figure 8. Histogram reorganization reassigns the pixel values of the Gaussian-like distribution ciphertext, thereby breaking the conventional distribution range of ciphertext values within [0,255]. After bit permutation, the resulting ciphertext exhibits stronger resistance to statistical analysis. Meanwhile, the encrypted noise-like ciphertext closely matches the optimal histogram distribution under the current embedding constraints and maintains high ciphertext quality. The following subsections present the fundamental principles of the embedding process, the implementation method and theoretical explanation of histogram shifting, and finally the bit permutation procedure under M-ary decomposition.

#### Principle of embedding

Here, we first introduce the embedding process, which facilitates a clearer understanding of the role of histogram reorganization described later. As shown in Figure 8, the embedding procedure primarily involves incorporating the four components of the noise-like ciphertext, obtained through M-ary decomposition, into the four frequency subbands of the cover image via GLSB embedding and correction. These four subbands are extracted by applying the integer discrete wavelet transform (DWT) to the cover image. In the following, we briefly present the formulas and principles of M-ary decomposition, GLSB embedding, and the correction method as proposed in Jing et al.^39^

##### M-ary decomposition

M-ary decomposition is a generalized form of binary decomposition. The key distinction is that binary decomposition is restricted to a base of 2, whereas M-ary decomposition allows an arbitrary base *M*. Since the cover image is divided into four parts, the noisy sample information is decomposed into four corresponding components. The decomposition and reconstruction formulas are given in Equations 22 and 23, respectively.

(Equation 22) $$\left\{\begin{array}{c} ca1=\;\text{floor}\;\left(\frac{NC}{B_{2}\times B_{3}\times B_{4}}\right)\\ ch1=\;\text{floor}\;\left(\frac{\;\mathrm{mod}\;\left(NC,B_{2}\times B_{3}\times B_{4}\right)}{B_{3}\times B_{4}}\right)\\ cv1=\text{floor}\;\left(\frac{\;\mathrm{mod}\;\left(NC,B_{3}\times B_{4}\right)}{B_{4}}\right)\\ cd1=\;\mathrm{mod}\;\left(NC,B_{4}\right) \end{array}\right)$$

(Equation 23) $$NC=ca1\times \left(B_{2}\times B_{3}\times B_{4}\right)+ch1\times \left(B_{3}\times B_{4}\right)+cv1\times B_{4}+cd1$$

Here, *ca*1, *ch*1, *cv*1, and *cd*1 denote the four components obtained through the decomposition. The basis for the decomposition is constrained by B_1_ ×B_2_ ×B_3_ ×B_4_ ≥ *NC*_max_, where *NC*_max_ denotes the maximum value within the value range of the noise-like ciphertext *NC*.

##### GLSB and correction

The GLSB embedding is derived from conventional LSB embedding, while the correction procedure is based on the 2^*k*^ correction method. The main advantage of this approach is that the modifications to the cover image can be inferred from the embedded values, allowing the ciphertext quality to be improved by controlling the embedded information. Taking the embedded *ca*1 component as an example, its embedding and subsequent correction processes are described by Equations 24 and 25, respectively.

(Equation 24) $$ca2=\;\mathrm{mod}\;\left(\;\mathrm{mod}\;\left(ca,B_{1}\right)+ca1,B_{1}\right)+\;\text{floor}\;\left(\frac{ca}{B_{1}}\right)\times B_{1}$$

(Equation 25) $$ca3=\left\{\begin{array}{c} ca2-B_{1}\;\;\text{if}\;\;ca2-ca>B_{1}/2\\ ca2+B_{1}\;\;\text{if}\;\;ca2-ca<-B_{1}/2\\ ca2\;\;\text{else}\; \end{array}\right)$$

Here, *ca* denotes one of the four components of the cover, *B*_1_ represents the least significant embedded bit corresponding to the decomposition basis of *ca*1, and *ca*3 denotes the corrected value. For example, when *ca* = 128, *ca*1 = 3, and *B*_1_ = 4, the embedded result obtained from Equation 24 is 131, which is subsequently corrected to 127 according to Equation 25. The extraction process is performed as described in Equation 26.

(Equation 26) $$ca1=\;\mathrm{mod}\;\left(\;\mathrm{mod}\;\left(ca3,\;B_{1}\right)-\;\mathrm{mod}\;\left(ca,\;B_{1}\right),B_{1}\right)$$

#### Histogram reorganization

##### Implementation details

Histogram reorganization aims to redistribute the Gaussian-like distribution ciphertext according to the optimal distribution required for embedding, thereby achieving the desired histogram shape. This process requires two inputs: the index *index*_*C*1_ of the histogram of the Gaussian-like distribution ciphertext sorted in descending order, and the index *index*_*tk*_ corresponding to the optimal histogram distribution under the current transformation kernel. The reorganization operation is expressed by Equations 27 and 28.

(Equation 27) $$index_{C1}=Dsort\left(hist\left(C1\right)\right)$$

(Equation 28) $$C_{2}\left(p,q\right)=\left\{\begin{array}{cc} {\text{index}}_{tk}\left(i\right), & \text{if}\;C_{1}\left(p,q\right)={\text{index}}_{c1}\left(i\right),\\ C_{1}\left(p,q\right), & \text{otherwise}, \end{array}\right)\;\forall \;\left(p,q\right),\;i=1,2,\ldots ,256$$

The histogram of an image is computed using the *hist*() function. Here, *p* and *q* represent the coordinate positions. The inverse of the shifting operation is given by Equation 29.

(Equation 29) $$C_{1}\left(p,q\right)=\left\{\begin{array}{cc} {\text{index}}_{c1}\left(i\right), & \text{if}\;C_{2}\left(p,q\right)={\text{index}}_{tk}\left(i\right),\\ C_{2}\left(p,q\right), & \text{otherwise}, \end{array}\right)\;\forall \;\left(p,q\right),\;i=1,2,\ldots ,256$$

##### Working principle of histogram reorganization and derivation of the optimal histogram distribution

By analyzing Equations 24 and 25, the change in amplitude before and after embedding can be determined and further expressed by Equation 30. Using the aforementioned example, with *ca* = 128, *ca*1 = 3, and *B*_1_ = 4, applying Equations 24 and 25 yields *ca*3 = 127. The difference between *ca*3 and *ca* is *ca*3 − *ca* = 127 − 128 = −1 = 3 − 4 = *ca*1 − *B*_1_. When *B*_1_ = 4, *ca*1 ranges over [0, 3], and *ca* is uniformly distributed. The relationship between the variation *v* of *ca* (according to Equation 30) and *ca*1 is summarized in Table 1. The first row of the table corresponds to the least significant digit of *ca* under the decomposition base *B*_1_ (values 0, 1, 2, 3, respectively), while the first column represents the value of *ca*1 (also 0, 1, 2, 3, respectively).

(Equation 30) $$v=\left\{\begin{array}{c} ca1\;\;\text{if}\;\;ca1\leq B_{1}/2\\ ca1-B_{1}\;\;\text{else}\; \end{array}\right)$$

**Table 1:** Cover image changes

	0	1	2	3
0	0	0	0	0
1	1	1	1	1
2	2	2	2	2
3	−1	−1	−1	−1

Optimal embedding performance is achieved when the distribution of the embedded information is concentrated near 0 or *B*_1_, which corresponds to the desired embedding pattern for each segmented region. In the spatial domain, each segmented region exhibits the same characteristics as the image as a whole; therefore, the determination of the optimal histogram distribution is relatively straightforward. In contrast, in the integer wavelet transform domain, variations in different frequency components influence the overall ciphertext in distinct ways. Table 2 presents the impact of modifying individual frequency components of the cover under the Haar transform kernel. For example, increasing the low-frequency component *ca* to *ca* + 1 and then applying the inverse transform results in a change magnitude of *mn* in both the ciphertext and the cover. By comparison, increasing the high-frequency component *cd* to *cd* + 1 leads to a change magnitude of 0.25× *mn*. It is noteworthy that, even within the same frequency band, applying identical modification amplitudes under different transform kernels may produce different levels of change in the cover. Moreover, when multiple frequency bands are modified simultaneously, the resulting changes are not linearly additive. For instance, increasing two frequency components by 1 yields the cover variations reported in Table 3. The results presented in Tables 2 and 3 correspond to idealized conditions, in which odd and even coefficients are assumed to be uniformly distributed within each band.

**Table 2:** Effects of varying frequencies on the cover

	ca	ch	cv	cd
0	0	0	0	0
1	1	0.5	0.5	0.25
2	2	1	1	0.5
3	3	1.5	1.5	0.75
4	4	2	2	1

**Table 3:** Effects of adding 1 to both frequencies on the cover

	ca	ch	cv	cd
ca	∼	1	1	1
ch	1	∼	0.75	0.625
cv	1	0.75	∼	0.5
cd	1	0.625	0.5	∼

By analyzing Tables 2 and 3, the top 256 entries with the smallest cover variations are selected and sorted in ascending order according to the magnitude of cover change. These entries serve as the indices *index*_*tk*_ corresponding to the optimal histogram distribution employed in histogram reconstruction. Once the transformation kernel is fixed, the associated *index*_*tk*_ is uniquely determined. The procedure for obtaining *index*_*tk*_ is summarized in Algorithm 1. For all transformation kernels, the corresponding *index*_*tk*_ and decomposition basis *B* are identified and recorded within the cryptographic system, enabling their direct invocation during the encryption process. This strategy substantially reduces both encryption and decryption times.

Algorithm 1 **Determination of the optimal histogram index indextk and the corresponding decomposition basis B = [B1, B2, B3, B4].**

**Input:** Integer wavelet transform kernel *TK*.

**Output:** *index*_*tk*_, *B* = [*B*_1_, *B*_2_, *B*_3_, *B*_4_].

1: Randomize each frequency-band parameter to ensure a uniform distribution of odd, even, positive, and negative values.

 *ca* = ⌊255 × *rand*(1024, 1024)⌋.

 *ch* = ⌊40 × *rand*(1024, 1024)⌋ − 20.

 *cv* = ⌊40 × *rand*(1024, 1024)⌋ − 20.

 *cd* = ⌊40 × *rand*(1024, 1024)⌋ − 20.

 *im* = *IDWT*(*ca*, *ch*, *cv*, *cd*, *TK*).

2: Modify each frequency band and evaluate the PSNR of the cover before and after modification.

 *num* = 1.

 *psnr*_*tem*_ = [].

 *tem* = [].

 for *i*1 = −*a*1: *a*1.

  for *i*2 = −*a*2: *a*2.

   for *i*3 = −*a*3: *a*3.

    for *i*4 = −*a*4: *a*4.

     *tem*(*num*, ) = [*i*1, *i*2, *i*3, *i*4].

     *rim* = *IDWT*(*ca* + *i*1, *ch* + *i*2, *cv* + *i*3, *cd* + *i*4, *TK*).

     *psnr*_*tem*_(*num*) = *psnr*(*im*, *rim*).

     *num* = *num* + 1.

    end.

   end.

  end.

 end.

3: Sort *psnr*_*tem*_ in descending order, extract the corresponding indices, and select the top 256 entries.

 *index* = *Dsort*(*psnr*_*tem*_).

 *tem*3 = *tem*(*index*(1 : 256), :).

4: Determine the decomposition basis and the lowest effective embedding bit *B* = [*B*_1_, *B*_2_, *B*_3_, *B*_4_].

 *B*_1_ = 2 × *max*(|*tem*3(:, 1)|) + 1.

 *B*_2_ = 2 × *max*(|*tem*3(:, 2)|) + 1.

 *B*_3_ = 2 × *max*(|*tem*3(:, 3)|) + 1.

 *B*_4_ = 2 × *max*(|*tem*3(:, 4)|) + 1.

5: Compute *index*_*tk*_ based on *B* and *tem*3.

 *tem*3(*tem*3(:, 1) < 0, 1) = *tem*3(*tem*3(:, 1) < 0, 1) + *B*_1_

 *tem*3(*tem*3(:, 2) < 0, 2) = *tem*3(*tem*3(:, 2) < 0, 2) + *B*_2_

 *tem*3(*tem*3(:, 3) < 0, 3) = *tem*3(*tem*3(:, 3) < 0, 3) + *B*_3_

 *tem*3(*tem*3(:, 4) < 0, 4) = *tem*3(*tem*3(:, 4) < 0, 4) + *B*_4_

 *index*_*tk*_ = *tem*3(:, 1)×(*B*_2_×*B*_3_×*B*_4_) + *tem*3(:, 2)×(*B*_3_×*B*_4_) + *tem*3(:, 3)×*B*_4_ + *tem*3(:, 4).

*rand*(*m*, *n*) generates an *m* × *n* matrix with uniformly distributed random values in [0, 1].

*psnr*(*x*, *y*) computes the peak signal-to-noise ratio between images *x* and *y*.

*IDWT*(*ca*, *ch*, *cv*, *cd*, *TK*) performs the integer discrete wavelet transform.

*Dsort*(*x*) sorts *x* in descending order and returns the corresponding indices.

*a*1, *a*2, *a*3, *a*4  denote the loop ranges. These ranges can be obtained according to the value of *B* given in effect of different transformation kernels on ciphertexts, yielding a corresponding initial value, which is then expanded by two values in each direction. Alternatively, they can be directly set to 128, though this would require more loop iterations.

**Figure 9:**
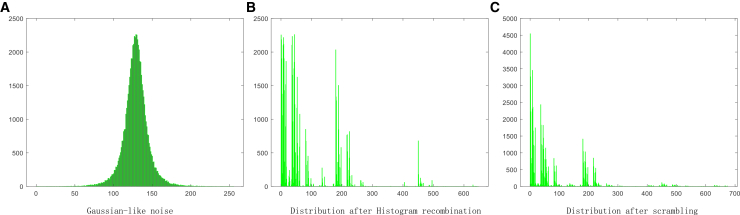
Histogram distribution change (A) Gaussian-like noise. (B) Distribution after histogram recombination. (C) Distribution after scrambling.

To illustrate the effectiveness of the proposed method, the “Woman” image from the standard test dataset (https://www.imageprocessingplace.com/root_files_V3/image_databases.htm) is employed as a representative example. Figure 9 presents the distributions of the noise-like components before and after histogram reconstruction. Quantitative analysis reveals that the PSNR of the ciphertext in the wavelet transform domain increases from approximately 45 to over 49 dB following histogram reconstruction. Similarly, the PSNR of the spatial-domain ciphertext improves from approximately 46 to over 51 dB. These experimental results demonstrate that histogram reconstruction significantly enhances the visual quality of the ciphertext, thereby improving the robustness of ciphertext transmission.

#### Bit scrambling under M-ary decomposition

Although the histogram of the Gaussian-like distribution ciphertext *C*1 is altered after histogram reorganization, the total number of values remains unchanged. For example, according to Equation 29, the number of elements equal to 129 in *C*1 becomes 13, while the overall number of elements is preserved. Using such a ciphertext directly as an intermediate representation may therefore raise security concerns. To address this issue, this paper introduces a shuffling strategy based on M-ary decomposition. Specifically, the Gaussian-like distribution ciphertext *C*1 is first decomposed into four components, *ca*1, *ch*1, *cv*1, and *cd*1, according to Equation 22. Each component then undergoes independent row and column shuffling. The low-frequency component *ca*1 is scrambled using Equation 31 to obtain *ca*2, and the same procedure is applied to the remaining frequency bands, yielding *ch*2, *cv*2, and *cd*2, respectively.

(Equation 31) $$\left\{\begin{array}{c} ca2\left(R1\left(i\right),:\right)=\text{circshift}\;\left(\left(ca1\left(R1\left(i\right),:\right),R2\left(i\right)\right)\right)\;\;\text{if}\;\;R1\left[i\right]\leq m\\ ca2\left(:,R1\left(i\right)\right)=\text{circshift}\;\left(\left(ca1\left(:,R1\left(i\right)\right),R2\left(i\right)\right)\right)\;\;\text{else}\; \end{array}\right)$$

**Figure 10:**
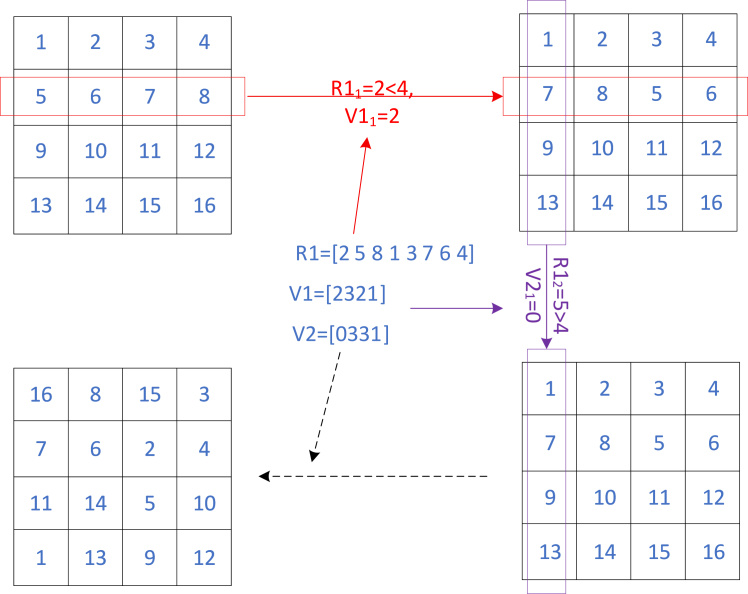
Randomly out-of-order cycle shuffle

The operator circshift denotes circular shifting, where *R*2 specifies the shift magnitude, *R*1 determines the selected row or column, and *mn* denotes the height and width of the reorganized Gaussian-like distribution ciphertext *C*1. Conventional circular permutation schemes typically follow a fixed row-then-column order, which may introduce predictable patterns during decryption and thereby weaken security. To overcome this limitation, this paper proposes a disordered circular shift scrambling algorithm that disrupts the scrambling sequence, increases computational complexity, and enhances security. The overall scrambling procedure is illustrated in Figure 10. Specifically, the sequence *R*1 determines which row or column to scramble. When *R*1 ≤ *m*, the *R*1-th row is circularly shifted to the left by *V*1 positions; when *R*1 > *m*, the (*R*1 − *m*)-th column is circularly shifted to the left by *V*2 positions. Subsequently, Equation 23 recombines the scrambled components to produce the final noise-like ciphertext *NC*. Under M-ary decomposition, this scrambling process simultaneously modifies both the position within the corresponding decomposition basis and the overall pixel values, as illustrated in Figure 9C, where the pixel value distribution is completely transformed. It is noteworthy that this scrambling operation does not degrade the quality of the ciphertext used for embedding. This is because, although the distribution of the overall noise-like ciphertext is altered, the statistical distribution of the decomposition values remains unchanged.

### Encryption and decryption process

**Figure 11:**
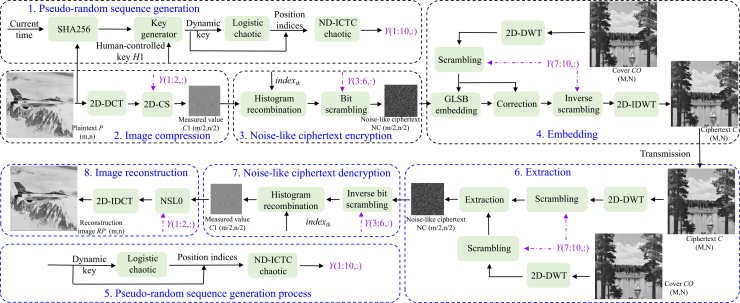
Encryption and decryption process The black dashed box denotes the encryption procedure, while the blue dashed box represents the decryption procedure.

For clarity, the image encryption procedure is described using grayscale plaintext and cover images in conjunction with spatial-domain embedding. At the encryption side, the plaintext image is first processed through compressive encryption and embedding to generate a visually meaningful ciphertext and a dynamic secret key, which are then transmitted to the decryption side via a public network or a secure channel. Upon reception, the ciphertext is subjected to extraction and decryption to recover the plaintext image. The overall encryption-decryption framework is illustrated in Figure 11.

When the plaintext is a color image, each Red-Green-Blue (RGB) channel is first compressed independently, and the resulting measurements are subsequently reorganized for further processing. For a color cover image, the three color channels are concatenated row by row to form a large grayscale image. In the case of spatial-domain embedding, the wavelet transform is replaced by spatial partitioning of the cover image. Specifically, the cover image is evenly divided into four regions according to spatial positions, namely the upper-left, upper-right, lower-left, and lower-right regions.

#### Encryption process

The proposed encryption scheme mainly consists of four stages: pseudorandom sequence generation, image compression, noise-like ciphertext encryption, and visually meaningful ciphertext embedding. The detailed procedures are described as follows.

##### Pseudorandom sequence generation

The pseudorandom sequences are mainly employed for generating the measurement matrix and controlling permutation operations. All the required sequences in this scheme are produced by the ND-ICNTC chaotic system. Since a total of ten independent sequences are required, a ten-dimensional chaotic system is directly constructed in this study. The initial conditions and position indices of the ND-ICNTC system are independently generated and used as secret keys, resulting in a total of 22 dynamic keys that need to be transmitted. Such a large key size is inconvenient for practical transmission. To address this issue, a logistic chaotic map is adopted to generate the initial states and position indices of the ND-ICNTC system. Consequently, only four secret keys are required for the two chaotic systems combined. The detailed procedure for generating the ten-dimensional chaotic sequences is described as follows.

Step 1: the dynamic key generation procedure is illustrated in Algorithm 2.

Algorithm 2 **Dynamic key generation**

**Input:** The plaintext P and the 256-bit human-controlled key *H*1 = [*h*1_1_, *h*1_2_, *…*, *h*1_256_]

**Output:** The initial value *x*_0_ and parameters *μ*, *μ*_1_, *μ*_2_

1: Calculate the Secure Hash Algorithm (SHA)256 value *H*2 = [*h*2_1_, *h*2_2_, *…*, *h*2_256_] of P.

2: Calculate the SHA256 value *H*3 = [*h*3_1_, *h*3_2_, *…*, *h*3_256_] of T.

3: The corresponding positions of H1, H2 and H3 are XOR to obtain a new 256-bit bit *H* = [*h*_1_, *h*_2_, *…*, *h*_256_].

4: Convert every eighth digit into a decimal sum according to the following equation $sha_{\mathrm{sum}}=\sum_{i=1}^{32}bin2dec\left(H_{\left(i-1\right)\times 8+1:i\times 8}\right)/256$.

5: Obtain the initial value *x*_0_ and the parameters *μ*, *μ*_1_, *μ*_2_.

 
$x_{0}=\left(\left(sha_{\mathrm{sum}}/32+bin2dec\left(H_{1:8}\right)/256\right)\right)mod1$

 
$\mu =\left(\left(sha_{\mathrm{sum}}/32+bin2dec\left(H_{10:18}\right)/256\right)\right)mod0.1+3.9$

 *μ*_1_ = (*sha*_*sum*_/32 + *bin*2*dec*(*H*_20:28_)/256) × 10 + 30.

 *μ*_2_ = (*sha*_*sum*_/32 + *bin*2*dec*(*H*_30:38_)/256) × 10 + 30.

Step 2: the initial value *x*_0_ and parameter *μ* are substituted into the logistic chaotic map defined in Equation 1, and the system is iterated 130 times to generate the sequence *X*. Subsequently, the subsequences *X*_*100:110*_ and *X*_*110:120*_ are substituted into Equation 2 for sorting and index extraction, respectively, yielding the index sequences *J* and *M*.

Step 3: the parameters *μ*_1_ and *μ*_2_, together with the initial values *X*_*120:130*_ and the indices *J* and *M*, are substituted into Equation 6. The system is then iterated *m* × *n* + 100 times. To eliminate the transient effects of chaos, the first 100 iterations are discarded, and the remaining outputs constitute the ten-dimensional chaotic sequence *Y*. Here, *m* and *n* denote the height and width of the plaintext image *P*, respectively.

##### Image compression

In this work, the two-dimensional compressive sensing (2DCS) framework proposed in Jing et al.^39^ is adopted. The plaintext image is first sparsified using the discrete cosine transform (DCT). To enhance sampling efficiency, the measurement matrix is optimized to allocate more sampling weights to the upper-left (low-frequency) components, which contain more significant image information. The overall procedure is summarized as follows.

Step 1: the optimization of the measurement matrix is defined by Equation 32, where orth(・) denotes the orthogonalization operation. Specifically, the first *m* × *m* × *cr* elements from the first dimension of the chaotic sequence *Y* are substituted into Equation 32 to generate the measurement matrix *ϕ*_1_. Similarly, the first *n* × *n* × *cr* elements from the second dimension of *Y* are substituted into Equation 32 to obtain *ϕ*_2_.

(Equation 32) $$\left\{\begin{array}{c} x^{\prime }=1-2\times \;\mathrm{mod}\;\left(x\times 10^{4},1\right)\\ \phi =reshape\left(x^{\prime },\left[cr\times m,m\right]\right)\\ \phi \left(:,1:cr\times m\right)=\phi \left(:,1:cr\times m\right)\times 5000\\ \phi =orth{\left(\phi^{T}\right)}^{T} \end{array}\right)$$

Step 2: the plaintext image *P* is sparsified using the DCT and subsequently compressed by applying the optimized measurement matrices *ϕ*_1_ and *ϕ*_2_, as expressed by

(Equation 33) $$X1=\phi_{1}*DCT\left(P\right)*\phi_{2}^{\text{T}}$$

Step 3: finally, quantization is performed on the compressed measurements to obtain the Gaussian-like distribution ciphertext *C*1:

(Equation 34) $$C1=\lfloor \frac{X1-\min_{X1}}{max_{X1}-min_{X1}}\times 255\rfloor$$

##### Noise-like ciphertext encryption

This stage mainly aims to further encrypt the measurement values *C*1 to enhance security and to generate a noise-like ciphertext with a distribution that is more suitable for the subsequent embedding stage. The detailed procedure is described as follows.

Step 1: the decomposition basis *B* and the optimal histogram index *index*_*tk*_ are determined according to the selected wavelet transform kernel *TK*.

Step 2: the Gaussian-like distribution ciphertext *C*1 and the optimal histogram index *index*_*tk*_ are substituted into Equations 27 and 28 to obtain the histogram-reorganized matrix *X*2.

Step 3: the reorganized matrix *X*2 is decomposed using the decomposition basis *B* according to Equation 22, yielding the four components *ca*1, *ch*1, *cv*1, and *cd*1.

Step 4: the third dimension of the chaotic sequence *Y*, i.e., *Y*(3, :), is substituted into the following equations to generate the cyclic shift control sequences *R*1 and *R*2. Subsequently, *R*1 and *R*2 are combined with *ca*1 in Equation 31 to produce the scrambled matrix *ca*2. In an analogous manner, the fourth to sixth dimensions of *Y* are employed to scramble *ch*1, *cv*1, and *cd*1, resulting in *ch*2, *cv*2, and *cd*2, respectively.

(Equation 35) $$\left\{\begin{array}{c} R1=mod\left(Y\left(3,1:mn\times cr\right),\left(m+n\right)\times cr\right)\\ R2=mod\left(Y\left(3,mn\times cr+1:2mn\times cr\right),n\right) \end{array}\right)$$

Step 5: finally, the scrambled components *ca*2, *ch*2, *cv*2, and *cd*2 are recombined via Equation 23 to generate the final noise-like ciphertext *NC*.

##### Meaningful ciphertext embedding

As introduced in noise-like ciphertext generation and embedding, the four components obtained from the M-ary decomposition of the noise-like ciphertext *NC* are embedded into four distinct frequency bands of the cover image using the GLSB embedding and correction strategy. Directly embedding these components into fixed spatial or frequency positions (e.g., the top-right region) may lead to an uneven distribution of embedded information, especially when the cover image is significantly larger than the payload, thereby increasing the risk of detection. To address this issue, an additional scrambling operation is incorporated during the embedding stage. This operation ensures a more uniform spatial distribution of the embedded information over the cover image while simultaneously introducing an extra layer of encryption, thereby further enhancing the overall security of the proposed scheme.

Step 1: the cover image *Co* is transformed using the integer wavelet transform to obtain its frequency-band components:

(Equation 36) ca,ch,cv,cd=DWT(Co,TK)

Step 2: considering that the cover image may be up to four times larger than the embedded information, a scrambling operation is applied to evenly distribute the embedding positions. This scrambling procedure is identical to the one described previously. Specifically, the last four dimensions of the chaotic sequence *Y* are employed to scramble *ca*, *ch*, *cv*, and *cd*, yielding the scrambled components *ca*3, *ch*3, *cv*3, and *cd*3, respectively.

Step 3: GLSB embedding and the corresponding correction are then performed between *ca*2 and *ca*3 using Equations 24 and 25 to obtain *ca*4. The same embedding and correction process is applied to the remaining components *ch*2, *cv*2, and *cd*2 with *ch*3, *cv*3, and *cd*3, resulting in *ch*4, *cv*4, and *cd*4, respectively.

Step 4: the inverse scrambling operation is applied to *ca*4, *ch*4, *cv*4, and *cd*4 to obtain *ca*5, *ch*5, *cv*5, and *cd*5. The inverse scrambling is achieved by using the negated shift parameter *R*2 and applying Equation 31 in reverse order with respect to the control sequence *R*1.

Step 5: finally, the meaningful ciphertext *C* is reconstructed by applying the inverse integer wavelet transform to *ca*5, *ch*5, *cv*5, and *cd*5:

(Equation 37) C=IDWT(ca5,ch5,cv5,cd5,TK).

#### Decryption process

In addition to the ciphertext, the encryption party must transmit the dynamic keys {*x*_0_, *μ*, *μ*_1_, *μ*_2_}, the wavelet transform kernel *TK*, and the permutation index *index*_*c*1_ of the measurement matrix histogram to the decryption party. These can be encrypted using a traditional asymmetric Rivest-Shamir-Adleman (RSA) algorithm and transmitted over a public network, or alternatively transmitted directly over a private network. If the encryption and decryption parties have not previously shared a cover image, the cover image must also be transmitted to the receiver. The decryption process consists of four stages: pseudorandom sequence generation, noise-like ciphertext extraction, noise-like ciphertext decryption, and plaintext reconstruction, as illustrated in the decryption portion of Figure 11. Each of these stages is described in detail as follows.

##### Pseudorandom sequence generation

The generation of the chaotic sequence *Y* follows the same procedure as that described in steps 2 and 3 of the encryption process.

##### Extraction of the noise-like ciphertext

Step 1: the decomposition basis *B* and the optimal histogram index *index*_*tk*_ are determined according to the wavelet transform kernel *TK*.

Step 2: both the cover image *Co* and the meaningful ciphertext *C* are transformed using the integer wavelet transform to obtain their corresponding frequency-band components:

(Equation 38) $$\left\{\begin{array}{c} ca,ch,cv,cd=IDWT\left(Co,TK\right)\\ ca5,ch5,cv5,cd5=IDWT\left(C,TK\right) \end{array}\right)$$

Step 3: the frequency-band components *ca*5, *ch*5, *cv*5, and *cd*5, together with *ca*, *ch*, *cv*, and *cd*, are scrambled using the last four dimensions of the chaotic sequence *Y*, yielding *ca*4, *ch*4, *cv*4, and *cd*4 and *ca*3, *ch*3, *cv*3, and *cd*3, respectively.

Step 4: the embedded information *ca*2 is extracted by substituting *ca*4 and *ca*3 into Equation 26. In the same manner, the remaining components *ch*2, *cv*2, and *cd*2 are recovered.

Step 5: finally, the noise-like ciphertext *NC* is reconstructed by combining *ca*2, *ch*2, *cv*2, and *cd*2 according to Equation 23.

##### Noise-like ciphertext decryption

Step 1: the third to sixth dimensions of the chaotic sequence *Y* are employed to perform inverse scrambling on *ca*2, *ch*2, *cv*2, and *cd*2, yielding the decomposed components *ca*1, *ch*1, *cv*1, and *cd*1, respectively.

Step 2: the histogram-reorganized matrix *X*2 is reconstructed by combining *ca*1, *ch*1, *cv*1, and *cd*1 according to Equation 23.

Step 3: the Gaussian-like distribution ciphertext *C*1 is recovered by applying inverse histogram reorganization to *X*2 using the optimal histogram index *index*_*tk*_, as expressed by Equation 29.

##### Plaintext reconstruction

Step 1: the two measurement matrices *ϕ*_1_ and *ϕ*_2_ are regenerated by following the same measurement matrix optimization procedure adopted during the compression stage.

Step 2: the sparsified coefficient matrix *T* is reconstructed using the NSL0 algorithm.^16^

Step 3: the final decrypted image *RP* is obtained by applying the inverse DCT to *T*:

(Equation 39) RP=IDCT(T).

## Results

All experiments were conducted on a personal computer equipped with 32 GB RAM, an Intel i7 processor, and Windows 11, using MATLAB R2018a as the simulation platform. The 256-bit user-defined secret key was set to 8d5ab8ba5340fce4420829ad5d12a0e45dacb0858544163d04c1d02b73e3697d. The compression ratio was fixed at 0.25, and the Haar wavelet was selected as the integer wavelet transform kernel. Three publicly available datasets were used for experimental evaluation: Computer Vision Group (CVG) (https://ccia.ugr.es/cvg/dbimagenes/c512.php), DIV2K (https://data.vision.ee.ethz.ch/cvl/DIV2K/), and BSDS100 (https://www2.eecs.berkeley.edu/Research/Projects/CS/vision/bsds/). The CVG dataset contains 64 images of size 512 × 512. From BSDS100, 100 images were center-cropped to 256 × 256. From DIV2K, 100 images from the test set were center-cropped to 1,024 × 1,024. If an image size was smaller than the specified resolution, bilinear interpolation was applied to rescale it to the required size. For grayscale images, the corresponding color images from the aforementioned datasets were converted to single-channel grayscale prior to processing. In the experiments, the noise-like ciphertext is denoted as NC, the visually meaningful ciphertext as C, the decrypted reconstructed image as RP, spatial-domain embedding as SP, and wavelet-domain embedding as WT.

### Encryption effect

**Figure 12:**
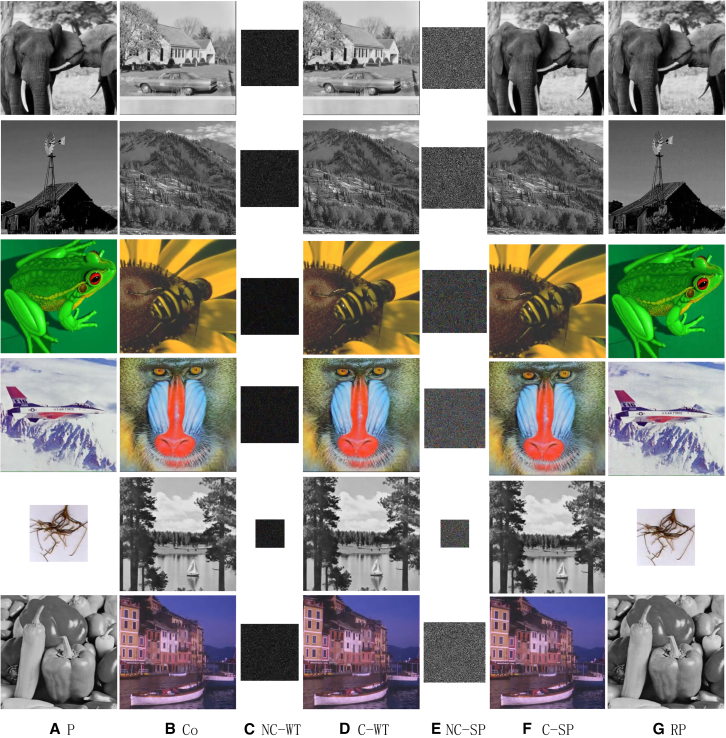
Encryption and decryption results (A) Plaintext image. (B) Cover image. (C) Noise-like ciphertext in the wavelet transform domain. (D) Meaningful ciphertext in the wavelet transform domain. (E) Noise-like ciphertext in the spatial domain. (F) Meaningful ciphertext in the spatial domain. (G) Decrypted image.

Figure 12 shows encryption and decryption results for representative images from the CVG dataset, with quantitative results in Table 4. Whether in spatial- or wavelet-domain embedding, the noise-like ciphertext appears noise-like with no discernible plaintext features, confirming effective concealment. The visually meaningful ciphertext is indistinguishable from the cover image, ensuring high quality and imperceptibility during transmission. Reconstructed images clearly reveal plaintext information, and quantitative results in Table 4 further confirm good encryption and decryption performance. Figure 12 also demonstrates that the algorithm handles grayscale and color images of various resolutions. Moreover, color images can be embedded into grayscale covers and vice versa, highlighting the scheme’s practicality and flexibility.

**Table 4:** Encryption and decryption results

Plaintext	Cover image	C				RP	
		WT		SP			
		PSNR	SSIM	PSNR	SSIM	PSNR	SSIM
elephant 512 × 512	house 512 × 512	49.2317	0.9222	51.1574	0.9883	34.1592	0.7901
windmill 512 × 512	utahmtn 512 × 512	49.3694	0.9958	51.4039	0.9970	30.0813	0.4940
frog 512 × 512 × 3	beeflowr 512 × 512 × 3	48.3594	0.9558	51.7800	0.9583	32.7206	0.9926
avion 512 × 512 × 3	baboon 512 × 512 × 3	50.2340	0.9996	52.1536	0.9997	31.4898	0.6886
raiz1 256 × 256 × 3	sailboat 512 × 512	51.0290	0.9875	52.3327	0.9909	29.7721	0.8346
peppers 512 × 512	portofino 512 × 512 × 3	53.7143	0.9997	55.8757	0.9998	32.9128	0.6286

### Effect of different transformation kernels on ciphertexts

**Figure 13:**
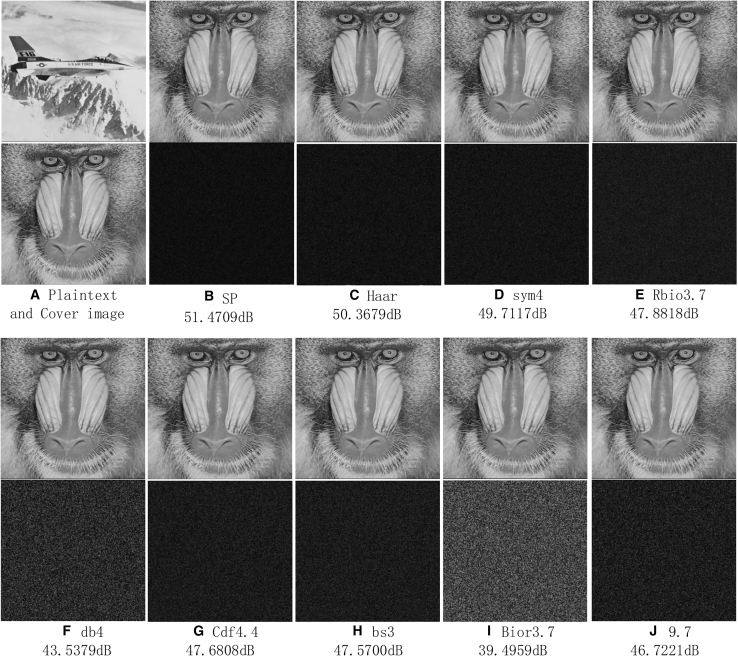
The effect of a transformation kernel on a ciphertext (A) Plaintext and Cover image. (B) SP PSNR = 51.4709dB. (C) Haar PSNR = 50.3679dB. (D) sym4 PSNR = 49.7117dB. (E) Rbio3.7 PSNR = 47.8818dB. (F) db4 PSNR = 43.5379dB. (G) Cdf4.4 PSNR = 47.6808dB. (H) bs3 PSNR = 47.5700dB. (I) Bior3.7 PSNR = 39.4959dB. (J) 9.7 PSNR = 46.7221dB.

Different transformation kernels have a significant impact on the resulting ciphertext, as they modify the extent of distortion introduced into the cover image by altering different frequency bands. Table 5 reports the ciphertext quality obtained on different datasets using various transformation kernels. In the experiments, the first image in each dataset is selected as the plaintext, while the subsequent images are sequentially used as cover images. As a result, 63, 99, and 99 ciphertext images are generated for the CVG, DIV2K, and BSDS100 datasets, respectively, with all images converted to grayscale. Figure 13 presents a visual comparison of ciphertexts produced using different transformation kernels when the plaintext is *avion* and the cover image is *baboon*. To facilitate observation, the difference images between the ciphertext and the corresponding cover are magnified by a factor of 50. The results in Figure 13 clearly indicate that the visual quality of the ciphertext varies considerably across different transformation kernels. The quantitative results in Table 5 further demonstrate that different transformation kernels lead to distinct optimal lowest embedding bits, and the corresponding PSNR and structural similarity index (SSIM) values of the ciphertexts also exhibit noticeable differences. Among the wavelet transform kernels, the Haar kernel achieves the highest ciphertext quality. Moreover, its performance in the spatial-domain embedding scenario is markedly superior to that obtained in the wavelet-transform-domain embedding. Consequently, all subsequent experiments adopt the Haar transform kernel and spatial-domain embedding. The results also confirm that different transformation kernels in Algorithm 1 yield different optimal histogram distribution indices and lowest embedding bits, indicating that the transformation kernel uniquely determines both the histogram index and the minimum embedding bit configuration.

**Table 5:** The effect of a transformation kernel on a ciphertext

TK	B	CVG		DIV2K		BSDS100	
		PSNR	SSIM	PSNR	SSIM	PSNR	SSIM
SP	4,4,4,4	51.0265	0.9948	51.4626	0.9964	51.0177	0.9972
Haar	3,5,5,9	48.8169	0.9856	49.3660	0.9925	49.0875	0.9961
Sym4	3,5,7,15	45.6379	0.9849	48.9183	0.9918	48.5970	0.9955
Rbio3.7	3,5,5,9	46.7550	0.9825	47.2763	0.9893	46.9507	0.9934
Db4	11,5,5,3	42.8011	0.9723	43.1682	0.9788	42.8279	0.9847
Cdf4.4	1,5,7,15	46.5489	0.9819	47.0305	0.9887	46.7638	0.9930
Bs3	1,5,7,15	46.5378	0.9819	47.0130	0.9887	46.7471	0.9930
Bior3.7	11,5,5,5	39.1732	0.9492	39.3434	0.9543	39.2314	0.9658
9.7	5,5,5,5	45.1370	0.9812	46.2017	0.9879	46.0019	0.9925

TK, wavelet transform kernel; B, the lowest embedded bits of the four bands and the decomposition base; SP, spatial domain embedding.

### Effect of different Gaussian-like distribution ciphertexts on ciphertexts

**Figure 14:**
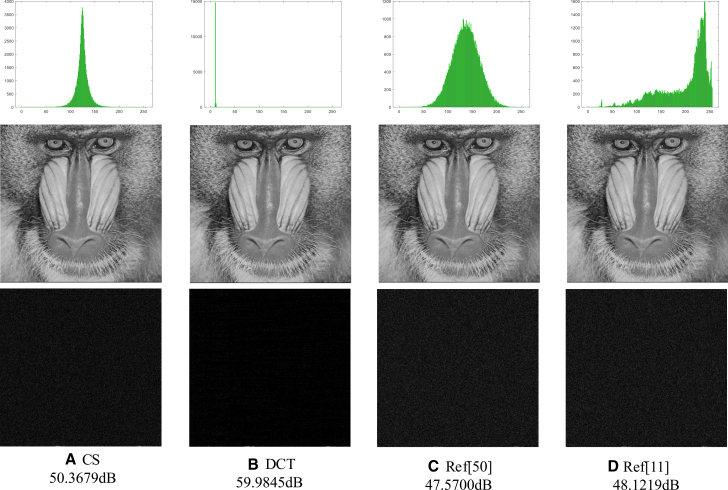
The effect of different Gaussian-like distribution ciphertexts on ciphertexts (A) CS PSNR = 50.3679 dB. (B) DCT PSNR = 59.984 5dB. (C) Refregier and Javidi^50^ PSNR = 47.5700 dB. (D) Wang and Zhang^11^ PSNR = 48.1219 dB.

Different distributions of Gaussian-like distribution ciphertext have a substantial impact on ciphertext quality. For this reason, multiple encryption algorithms are employed to generate Gaussian-like distribution ciphertexts and to evaluate their influence on the quality of meaningful ciphertexts. Existing approaches for producing Gaussian noise-like ciphertexts include optical image encryption methods,^8^^,^^50^ deep neural network-based schemes,^11^ and compressed sensing, as adopted in this paper. Table 6 compares the effects of four different Gaussian-like distribution ciphertexts on the resulting ciphertext quality using the Haar transform kernel. Figure 14 further illustrates the corresponding Gaussian-like histograms and ciphertexts when the plaintext is *avion* and the cover image is *baboon*. Here, “CS” denotes the compressed sensing-based scheme used in the proposed encryption algorithm, in which the cosine transform retains the upper-left quarter of the coefficients. For the other two algorithms, in order to achieve the same embedding capacity, the plaintext is uniformly downsampled to one-quarter of its original size. The results indicate that a more concentrated Gaussian-like histogram, corresponding to a smaller variance, leads to higher ciphertext quality. Therefore, by reducing the variance of the Gaussian-like distribution ciphertext, the proposed algorithm is able to further enhance the quality of the resulting meaningful ciphertext.

**Table 6:** The effect of different Gaussian-like distribution ciphertexts on ciphertexts

Method	CVG		DIV2K		BSDS100	
	PSNR	SSIM	PSNR	SSIM	PSNR	SSIM
CS	48.8169	0.9856	49.3660	0.9925	49.0875	0.9961
DCT	52.9264	0.9890	58.5166	0.9967	48.6292	0.9986
Refregier and Javidi^50^	41.5189	0.9659	41.7494	0.9718	41.5828	0.9798
Wang and Zhang^11^	41.3851	0.9650	41.4314	0.9699	41.4522	0.9791

### Conventional experimental analysis

This subsection presents a comprehensive evaluation of the proposed scheme using conventional security experiments, including statistical property analysis, key sensitivity, key space analysis, plaintext sensitivity, robustness, encryption and decryption efficiency, and resistance to chosen-plaintext attacks. The color images *avion* and *baboon* shown in Figure 12 are selected as the plaintext and cover images, respectively. All experiments are conducted using the Haar transform kernel in the wavelet transform domain for embedding. To verify the security and practicality of the proposed algorithm, we compared it with several state-of-the-art methods,^25^^,^^26^^,^^27^^,^^30^^,^^39^^,^^51^ all of which produce visually meaningful ciphertext through compressive sensing, encryption, and embedding operations. The source codes for Chen et al.,^25^^,^^27^^,^^51^ Shiwei and Jianjun,^30^ and Jing et al.^39^ were provided by the authors. For Yang et al.,^21^ we implemented the algorithm based on the description in the paper, and the simulation results were verified against the original publication to ensure consistency. Jing et al.^39^ also employ wavelet transform for embedding.

#### Key sensitivity

**Figure 15:**
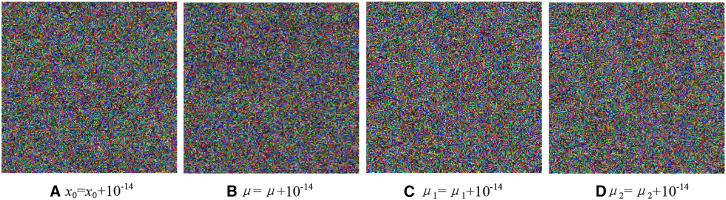
Decrypted image with slightly changed key (A) *x*_0_ = *x*_0_ + 10^-14^. (B) *μ* = *μ* + 10^-14^. (C) *μ*_1_ = *μ*_1_ + 10^-14^. (D) *μ*_2_ = *μ*_2_ + 10^-14^.

In the proposed scheme, the parameters and initial conditions of the chaotic system jointly constitute the dynamic secret keys. To evaluate the key sensitivity of the encryption algorithm, a tiny perturbation is introduced into the dynamic keys. Specifically, extremely small numerical variations are added to each of the four dynamic keys, and the corresponding decryption results are illustrated in Figure 15.

As shown in Figure 15, even an extremely minor modification to the key parameters results in a completely unintelligible decrypted image, from which no meaningful plaintext information can be recovered. This demonstrates that the proposed encryption scheme exhibits high sensitivity to key variations. Consequently, the algorithm can effectively resist brute-force attacks and key-related attacks, ensuring a high level of cryptographic security.

#### Key space

Among various attack methods, brute-force attack is the most common and simplest to execute. In this attack mode, the adversary exhaustively searches all possible keys in the key space. Therefore, an encryption algorithm must have a key space of at least 2^128^ to ensure security.^52^ The proposed encryption scheme employs four dynamic keys: the initial value *x*_0_ and control parameter *μ* of the logistic map, as well as the parameters *μ*_1_ and *μ*_2_ of the ND-ICNTC chaotic system. Based on the key sensitivity analysis discussed earlier, each key parameter exhibits high sensitivity at a precision of 10^14^. Consequently, the total key space of the proposed scheme is estimated to be 10^14×4^ ≈ 2^186^, which is sufficient to resist brute-force attacks. Furthermore, the proposed scheme can additionally use the initial values and position indices of the ND-ICNTC chaotic system generated by the logistic map as extra key parameters. This mechanism greatly expands the effective key space, further enhancing the security level of the encryption algorithm.

#### Robustness analysis

**Figure 16:**
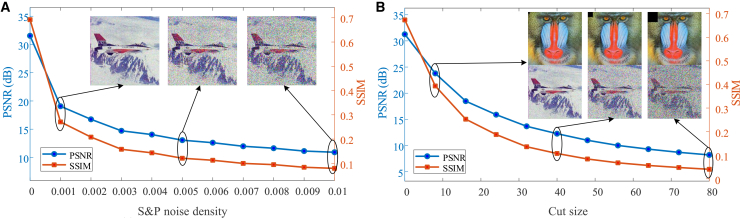
Decrypted image under ciphertext data loss and noise pollution (A) S&P noise density. (B) Cut size.

In practical communication scenarios, ciphertext may suffer from partial data loss or noise contamination during transmission. Therefore, an effective encryption scheme should possess sufficient robustness to ensure that the original plaintext information can still be recovered without complete destruction under moderate distortion. Figure 16 presents the decryption results for ciphertext subjected to different types and intensities of degradation, including data loss and noise contamination. As shown in Figure 16, as ciphertext degradation becomes more severe, both the PSNR and SSIM of the decrypted images decrease, and the visual quality deteriorates accordingly. Nevertheless, the main visual content of the plaintext remains recoverable, with key information still clearly discernible. These results demonstrate that the proposed encryption scheme exhibits satisfactory robustness against common transmission impairments, highlighting its practicality and applicability in real-world communication environments.

#### Statistical characteristic analysis

**Figure 17:**
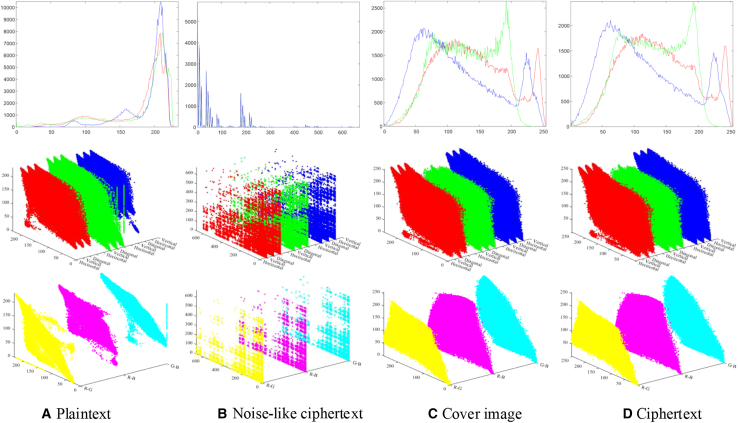
Statistical characteristic (A) Plaintext. (B) Noise-like ciphertext. (C) Cover image. (D) Ciphertext.

Traditional noise-like image encryption algorithms aim to eliminate the statistical characteristics of the plaintext to resist statistical attacks. In contrast, visually meaningful ciphertext encryption schemes require that the statistical properties of the ciphertext remain as consistent as possible with those of the cover image, so that the ciphertext remains visually indistinguishable from the cover image during transmission. To demonstrate that the proposed scheme effectively destroys the statistical correlation of the plaintext while preserving the statistical properties of the cover image, we conducted several analyses, including histogram distribution, adjacent pixel correlation, and RGB channel correlation. The results of our algorithm are shown in Figure 17. To further illustrate the advancement of the proposed algorithm, Table 7 presents a comparison of statistical properties between noise-like ciphertext and visually meaningful ciphertext for different algorithms. In the fourth column, P & C denote the plaintext and cover image, respectively, and CC denotes the correlation coefficient.

**Table 7:** Comparison of statistical characteristic among different algorithms

Reference			P & C	Yang et al.^26^	Jing et al.^39^	Shiwei and Jianjun^30^	Chen et al.^25^	Chen et al.^51^	Chen et al.^27^	Our
NC	Pixel value range		[0.255]	[0.255]	[0.269]	[0.255]	[0,64]	[0.255]	[0.255]	[0.674]
	Information entropy		6.5768	5.7387	6.4234	5.6671	5.9998	7.9948	7.9991	5.5751
	CC	Horizontal	0.9648	0.0001	0.0079	0.0037	−0.1567	−0.0005	0.0013	−0.0014
		Vertical	0.9533	0.0003	0.0039	−0.0031	−0.0029	−0.0038	−0.0003	−0.0017
		Diagonal	0.9272	0.0003	0.0002	0.0051	0.0007	−0.0018	−0.0009	0.0005
		R-G	0.9212	0.0018	−0.0066	0.0023	0.0032	−0.0018	−0.0002	−0.0012
		R-B	0.8410	−0.0013	−0.0002	−0.0053	0.0038	−0.0016	−0.0023	−0.0001
		G-B	0.9380	0.0004	0.0064	−0.0061	0.0036	0.0032	0.0036	0.0021
		Mean(abs())	0.92425	0.0007	0.0042	0.0043	0.0285	0.0021	0.0014	0.0012
C	Information entropy		7.6444	7.6040	7.6459	7.7637	7.7657	7.7632	7.7639	7.6451
	CC	Horizontal	0.8986	0.8981	0.8984	0.8983	0.8973	0.9133	0.8982	0.8984
		Vertical	0.8373	0.8368	0.8371	0.8370	0.8361	0.8539	0.8369	0.8372
		Diagonal	0.8097	0.8092	0.8095	0.8093	0.8085	0.8177	0.8093	0.8095
		R-G	0.3565	0.3558	0.3563	0.3564	0.3559	0.3516	0.3563	0.3564
		R-B	0.1237	0.1238	0.1235	0.1238	0.1235	0.1204	0.1236	0.1236
		G-B	0.8074	0.8070	0.8071	0.8071	0.8061	0.8079	0.8069	0.8072
		Mean(abs(C-Ref))	0	0.00045	0.00022	0.00025	0.00097	0.00800	0.00033	0.00015

As shown in Figure 17, the noise-like ciphertext exhibits a pixel value distribution completely different from that of the plaintext, indicating that the statistical structure of the plaintext has been thoroughly destroyed. The statistical distributions of the visually meaningful ciphertext and the cover image are indistinguishable in terms of both histogram shape and pixel correlation, demonstrating that the ciphertext retains the statistical characteristics of the cover image. From Table 7, it can be observed that although the IE of the noise-like ciphertext is not very high, it achieves a low correlation coefficient. This is mainly because the pixel value range of the noise-like ciphertext in our algorithm exceeds the normal range by more than a factor of two. Moreover, when encountering such an anomalous distribution, attackers are more likely to regard it as invalid or misleading data, thereby increasing the difficulty of cryptanalysis. Even in the case of chosen-ciphertext attacks, the large pixel value range significantly expands the attack space, leading to a substantial increase in the complexity and time cost of the attack. For the visually meaningful ciphertext, the results obtained by our algorithm are the closest to those of the cover image in terms of both IE and correlation. This further confirms that the proposed scheme generates high-quality visually meaningful ciphertext that is difficult for attackers to detect during transmission, thus providing strong resistance against statistical analysis attacks.

#### Plaintext sensitivity analysis

In traditional noise-like encryption, high ciphertext sensitivity to plaintext changes is crucial for resisting differential attacks. For visually meaningful ciphertext encryption, however, maintaining visual consistency with the cover image is the goal; excessive sensitivity is undesirable as it may impair ciphertext visual quality. Table 8 reports the impact of slight plaintext variations on the ciphertext in our algorithm, quantified using the number of pixels change rate (NPCR), unified average changing intensity (UACI), and SSIM. Table 9 presents the ciphertext changes for different algorithms when the first pixel of the plaintext is incremented by 1.

**Table 8:** Sensitivity analysis of plaintext

Change the way	NC			C		
	NPCR	UACI	SSIM	NPCR	UACI	SSIM
(1.1)+	0.9683	0.1246	−0.0020	0.6070	0.0030	0.9992
(128,512)−	0.9653	0.1173	−0.0004	0.5943	0.0028	0.9993
(1.1) ↔(128,512)	0.9670	0.1193	−0.0001	0.5916	0.0028	0.9993

**Table 9:** Comparison of plaintext sensitivity among different algorithms

Reference		Yang et al.^26^	Jing et al.^39^	Shiwei and Jianjun^30^	Chen et al.^25^	Chen et al.^51^	Chen et al.^27^	Our
NC	NPCR	0.9766	0.9885	0.9763	0.9846	0.9959	0.9961	0.9683
	UACI	0.0653	0.2684	0.0837	0.3383	0.3283	0.3356	0.1246
	SSIM	0.0587	−0.0029	0.0768	0.0784	0.0086	0.0022	−0.0020
C	NPCR	0.6534	0.7062	0.6097	0.6988	0.9039	0.7501	0.6070
	UACI	0.0041	0.0041	0.0036	0.0072	0.0129	0.0049	0.0030
	SSIM	0.9985	0.9987	0.9965	0.9866	0.9596	0.9946	0.9992

In Table 8, the notations (1,1)+ and (128,512)− denote increasing the pixel value at position (1,1) by 1 and decreasing the pixel value at position (128,512) by 1, respectively. The notation (1,1) ↔(128,512) indicates that the pixel values at positions (1,1) and (128,512) are exchanged. As shown in Table 8, the noise-like ciphertext exhibits a relatively low UACI under all three plaintext modification types. This is mainly because compressive sensing is employed as the diffusion operation without additional diffusion steps, resulting in concentrated measurement values. Nevertheless, the NPCR values are close to 1, and the SSIM values are close to 0. This indicates that even extremely small plaintext changes induce significant and unpredictable alterations in the noise-like ciphertext. For the visually meaningful ciphertext, although the NPCR reaches approximately 0.6, the UACI is close to 0 and the SSIM is very close to 1. This suggests that while the ciphertext changes in response to plaintext perturbations, the magnitude of change remains relatively small. This is primarily attributed to the histogram reorganization in the encryption process, which minimizes modifications to cover pixels during embedding. Consequently, the visually meaningful ciphertext maintains high visual similarity with the cover image. From Table 9, it can be observed that the NPCR and UACI of our noise-like ciphertext are weaker than those of algorithms^25^^,^^27^^,^^51^ that incorporate pixel value diffusion. This is because we adopt an approach similar to Yang et al., Shiwei and Jianjun, and Jing et al.,^21^^,^^30^^,^^39^ where compressive sensing directly serves as the diffusion operation, unlike Chen et al.,^25^^,^^27^^,^^51^ which introduce an additional diffusion step. Compared with Yang et al. and Shiwei and Jianjun,^21^^,^^30^ our algorithm achieves better UACI and SSIM. This improvement is mainly due to the bit-level scrambling operation, which alters the pixel values of the noise-like ciphertext, resulting in larger differences in pixel values at the same positions before and after plaintext changes. This further demonstrates the effectiveness of the proposed bit-level scrambling. The lowest SSIM indicates that the two ciphertexts in our scheme exhibit lower structural similarity, while the relatively high NPCR and UACI values reflect strong plaintext sensitivity and robust resistance to differential attacks. For the visually meaningful ciphertext, our algorithm achieves the lowest NPCR and UACI and the highest SSIM, indicating that plaintext variations have a minimal impact on the ciphertext. This indirectly reflects that the proposed meaningful ciphertext maintains high consistency with the cover image, demonstrating superior ciphertext quality.

#### Chosen-plaintext and chosen-ciphertext attack analyses

**Figure 18:**
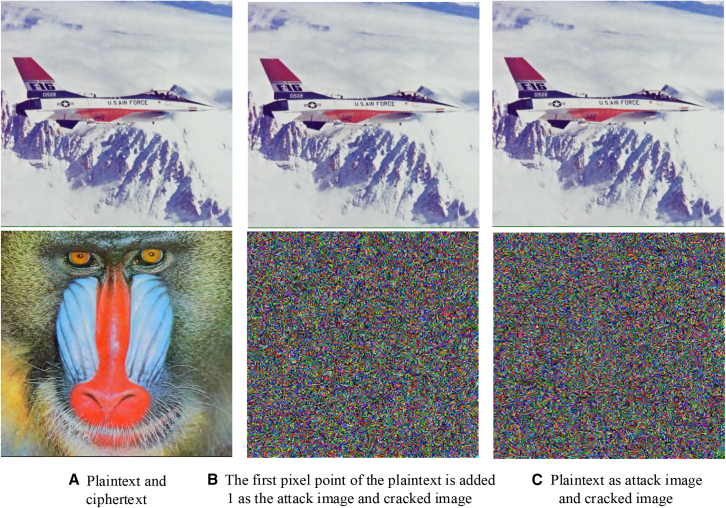
Choice of plaintext attack (A) Plaintext and ciphertext. (B) The first pixel point of the plaintext is added 1 as the attack image and cracked image. (C) Plaintext as attack image and cracked image.

Chosen-plaintext attack (CPA) and chosen-ciphertext attack (CCA) are among the most severe threats to encryption systems, and many image encryption schemes have been broken by CPA.^53^^,^^54^^,^^55^ The root cause is that the equivalent keystream is plaintext independent, allowing attackers to recover it using specific plaintexts under a fixed keystream. To address this, we generate chaotic initial values and parameters using the SHA hash of the plaintext, similar to Feng et al.,^52^ making the keystream plaintext dependent. Additionally, we incorporate the SHA hash of the current time, ensuring that even identical plaintexts produce different keystreams across encryption sessions—achieving a one-time one-key effect. To further verify the algorithm’s resistance to chosen-plaintext and chosen-ciphertext attacks, we assume that an attacker can recover the keystream corresponding to a chosen plaintext in a single encryption attempt. We select two types of plaintext as attack images: (1) a slightly modified version of the original plaintext and (2) the original plaintext itself. The corresponding keystreams are derived and then used to decrypt the original ciphertext. The decrypted images are shown in Figure 18.

As shown in Figure 18, all decrypted outputs are noise-like images revealing no meaningful information. Even in the extreme case where the attack image is identical to the original plaintext, the attack fails; in other scenarios, the probability of success is even lower. This robustness is primarily attributed to the plaintext-dependent and time-dependent dynamic key generation mechanism. Consequently, even when the attack image exactly matches the original plaintext, the dynamic keys used in the previous encryption process cannot be reproduced. Thus, the system achieves a one-time one-key encryption effect, greatly enhancing its resistance to both CPA and CCA and ensuring the security of the encryption algorithm.

#### Encryption and decryption efficiency

An effective image encryption algorithm must not only provide a high level of security but also achieve satisfactory computational efficiency in both encryption and decryption processes. To evaluate the efficiency of the proposed scheme, Table 10 presents the encryption and decryption times of our algorithm for images of different sizes, and Table 11 provides the encryption and decryption times of different algorithms for plaintext images of size 512 × 512 × 3.

**Table 10:** The encryption and decryption time (unit: S)

Image size	Compression and encryption	Embed	Extract	Reconstruction and decryption
512 × 512	0.2631	0.1366	0.0743	0.2648
512 × 512 × 3	0.5185	0.2706	0.1979	0.6641
1024 × 1024 × 3	1.6790	0.8544	0.6881	2.3662

**Table 11:** Encryption and decryption times of different algorithms (unit: S)

Reference	Yang et al.^26^	Jing et al.^39^	Shiwei and Jianjun^30^	Chen et al.^25^	Chen et al.^51^	Chen et al.^27^	Our
Compression and encryption	0.5568	0.5251	0.5226	0.2851	3.4236	3.2615	0.5185
Embed	0.3568	0.2610	0.9329	0.2860	0.0551	0.0768	0.2706
Extract	0.2519	0.1744	0.4871	0.0783	0.0322	0.0582	0.1979
Reconstruction and decryption	0.7845	0.6663	4.9755	0.1813	0.4292	0.3875	0.6641
All	1.9500	1.6268	6.9181	0.8307	3.9401	3.7840	1.6511

As shown in Table 10, the proposed algorithm maintains relatively fast performance across all tested image sizes. As expected, computational cost increases with image resolution due to the larger amount of data involved. Among all processing stages, the reconstruction and decryption stages are the most time-consuming, primarily because they involve inverse compressive sensing reconstruction. The space complexity of a single reconstruction iteration reaches *O*(*M*^3^), whereas other operations such as scrambling and embedding have complexities of only *O*(*M*^2^), where the image height and width are both *M*. Notably, replacing compressive sensing with alternative methods for generating Gaussian-like distribution ciphertext, as described in effect of different Gaussian-like distribution ciphertexts on ciphertexts, could further reduce decryption time by alleviating the computational burden of the reconstruction stage. Compared with other algorithms in Table 11, the total encryption and decryption time of our algorithm is comparable to that of Yang et al., Chen et al., and Jing et al.,^21^^,^^25^^,^^39^ all of which are relatively fast. The slower performance of Shiwei and Jianjun^30^ is mainly due to their sparse, scrambling, and compression approach, which requires numerous iterations during reconstruction. In contrast, our algorithm, along with Yang et al. and Jing et al.,^21^^,^^39^ employs two-dimensional compressive sensing with increased weights on the top-left corner of the measurement matrix. This design better samples the low-frequency information corresponding to the top-left corner of the sparse image, allowing plaintext reconstruction with only a few iterations and thus reducing the most time-consuming reconstruction step. Algorithms^25^^,^^27^^,^^51^ use deep networks for reconstruction; their end-to-end architecture requires no iteration, resulting in shorter reconstruction times. However, Chen et al.^27^^,^^51^ employ meta-learning to update the measurement matrix, which incurs high computational cost during compression. The algorithm in Chen et al.^25^ directly utilizes a learned measurement matrix and performs reconstruction with only a few convolutional layers, which significantly improves reconstruction speed and results in fast encryption and decryption. However, such a fixed measurement matrix offers weak resistance against chosen-plaintext and chosen-ciphertext attacks. Overall, the experimental results demonstrate that the computational efficiency of the proposed encryption scheme is comparable to that of existing fast algorithms, making it suitable for practical applications.

### Visually secure analysis

In visually meaningful ciphertext encryption, avoiding transmission detection is the primary goal, making ciphertext quality critical. To demonstrate our algorithm’s superiority, we compare it not only with the methods in conventional experimental analysis but also with two wavelet-domain algorithms known for high ciphertext quality^19^^,^^21^ and the pioneering meaningful ciphertext work.^12^ All three were implemented based on their papers and verified against the original results. Note that Bao and Zhou^12^ lack a noise-like encryption stage; we therefore adopted the noise-like component from Yang et al.^19^ for comparison. Additionally, the noise-like schemes in Yang et al.^19^^,^^21^ have no compression stage. For a fair embedding capacity comparison when evaluating ciphertext quality and steganalysis resistance, plaintext images in these two schemes were downsampled 4× via bilinear interpolation, ensuring comparable embedded information across all methods.

#### Meaningful ciphertext quality

**Figure 19:**
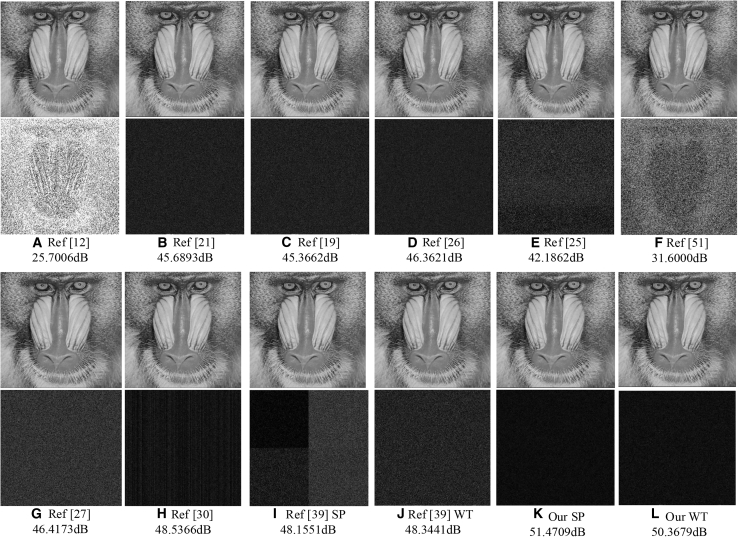
Different algorithms for meaningful ciphertexts (A) Bao and Zhou^12^ PSNR = 25.7006 dB. (B) Yang et al.^21^ PSNR = 45.6893 dB. (C) Yang et al.^19^ PSNR = 45.3662 dB. (D) Yang et al.^26^ PSNR = 46.3621 dB (E) Chen et al.^25^ PSNR = 42.1862 dB. (F) Chen et al.^51^ PSNR = 31.6000 dB. (G) Chen et al.^27^ PSNR = 46.4173 dB. (H) Shiwei and Jianjun^30^ PSNR = 48.5366 dB. (I) Jing et al.^39^ SP PSNR = 48.1551 dB. (J) Jing et al.^39^ WT PSNR = 48.3441 dB. (K) Our SP PSNR = 51.4709 dB. (L) Our WT PSNR = 50.3679 dB.

Table 12 presents a quantitative comparison of meaningful ciphertext quality among different algorithms across three benchmark datasets, while Figure 19 provides a visual comparison of ciphertexts obtained when the plaintext is avion and the cover image is baboon.

**Table 12:** Different algorithms have meaningful ciphertext quality

Ref.	CVG		DIV2K		BSDS100	
	PSNR	SSIM	PSNR	SSIM	PSNR	SSIM
Bao and Zhou^12^	27.2053	0.6928	27.3725	0.7106	26.4567	0.7364
Yang et al.^21^	43.9050	0.9747	44.5512	0.9835	44.7275	0.9911
Yang et al.^19^	43.7427	0.9747	44.4184	0.9832	44.5159	0.9904
Yang et al.^26^	45.5270	0.9875	45.8678	0.9895	45.8854	0.9927
Chen et al.^25^	42.1772	0.8718	42.1785	0.8571	42.1778	0.9145
Chen et al.^51^	27.3012	0.7295	27.7179	0.7461	28.4150	0.8302
Chen et al.^27^	46.3726	0.9896	46.3892	0.9919	46.3795	0.9953
Shiwei and Jianjun^30^	47.8863	0.9257	48.4719	0.9257	47.6042	0.9568
Jing et al.^39^ SP	47.9942	0.9910	48.2018	0.9927	47.9601	0.9949
Jing et al.^39^ WT	47.5137	0.9838	48.0267	0.9907	47.6221	0.9945
Our SP	51.0265	0.9948	51.4626	0.9964	51.0177	0.9972
Our WT	48.8169	0.9856	49.3660	0.9925	49.0875	0.9961

In Figure 19, the difference image between the ciphertext and the cover produced by the proposed scheme appears significantly darker than those generated by the compared methods, indicating a smaller distortion introduced during embedding. The quantitative results reported in Table 12 further corroborate this observation. For all three datasets, the proposed method consistently achieves the highest PSNR and SSIM values under both spatial-domain (SP) and wavelet-transform-domain (WT) embedding settings. Notably, the PSNR of the proposed scheme exceeds that of the best-performing existing methods by approximately 3 dB in the spatial domain and 1.5 dB in the wavelet transform domain, respectively, indicating a significant improvement in ciphertext imperceptibility. Meanwhile, the consistently high SSIM values demonstrate that the structural similarity between the ciphertext and the cover image is well preserved. Overall, both visual and numerical evaluations confirm that the proposed histogram reorganization-based meaningful encryption scheme produces ciphertexts with significantly higher visual quality than existing approaches, thereby enhancing the security and practicality of ciphertext transmission.

#### Steganalysis

Steganalysis is employed to evaluate the security of meaningful ciphertext images and constitutes a critical component in the assessment of meaningful ciphertext image encryption schemes. Specifically, steganalysis aims to quantify the detectability of meaningful ciphertext images by measuring the ability of steganalysis tools to distinguish them from genuine cover images. In general, mainstream steganalysis techniques can be broadly classified into two categories: traditional statistical feature-based methods and more recent approaches based on deep learning models.

##### Traditional steganalysis

**Figure 20:**
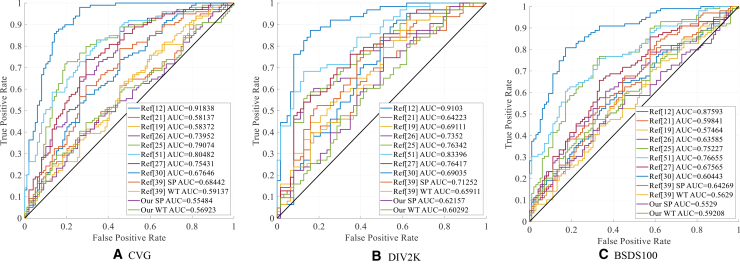
Traditional steganalysis (A) CVG. (B) DIV2K. (C) BSDS100.

StegExpose^56^ is an open-source steganalysis tool that integrates four widely used statistical detection methods, including principal-component analysis, sample pair analysis, the chi-squared test, and Regular-Singular (RS) analysis. Figure 20 presents the receiver operating characteristic (ROC) curves and the corresponding area under the ROC curve (AUC) values obtained using StegExpose for different encryption algorithms across multiple datasets.

As illustrated in Figure 20, for both spatial-domain embedding and wavelet-transform-domain embedding, the ROC curves produced by the proposed algorithm closely follow the diagonal line across all datasets, with AUC values approaching the ideal value of 0.5. This indicates that the meaningful ciphertexts generated by the proposed scheme are statistically indistinguishable from their corresponding cover images, thereby demonstrating strong resistance to traditional statistical steganalysis.

Compared with existing algorithms, the proposed method achieves superior steganalysis performance on nearly all datasets. Except for a slightly higher AUC observed for the wavelet-transform-domain embedding on the BSDS100 dataset when compared with Yang et al.^21^ and Jing et al.,^39^ the proposed approach consistently yields lower or comparable AUC values. Notably, the spatial-domain embedding strategy exhibits more pronounced advantages over competing methods. This is primarily because spatial-domain embedding directly reorganizes the histogram of the cover image with fine-grained control over pixel-level modifications, thereby better preserving first-order statistical features that are heavily exploited by traditional steganalysis tools. Overall, these results confirm that the proposed algorithm, particularly when employing spatial-domain embedding, provides strong robustness against traditional steganalysis attacks.

##### Deep learning steganalysis

**Figure 21:**
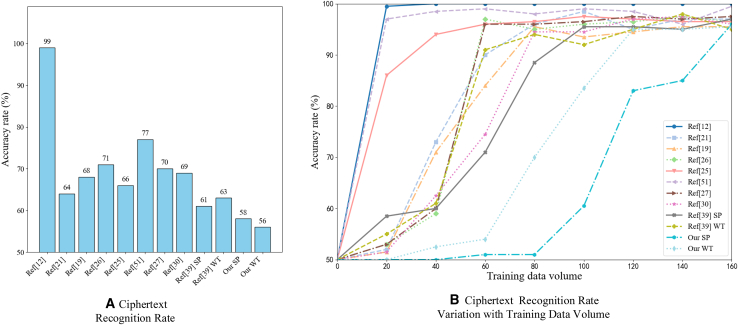
Deep learning steganalysis (A) Ciphertext recognition rate. (B) Ciphertext recognition rate variation with training data volume.

To further evaluate the resistance of visually meaningful ciphertext to steganalysis, two complementary deep-learning-based strategies were employed, both using SRNet^57^ as the steganalysis model. The first strategy used a model trained on a public dataset to test the detectability of ciphertext generated by different encryption algorithms. The training set consisted of cover-stego pairs (with random numbers as embedded data) generated by the Wavelet Obtained Weights (WOW) algorithm^58^ at an embedding rate of 0.5 bpp on the BOSSbase 1.01 dataset.^59^ The test set for each algorithm comprised stego images generated by encrypting the first 100 images of the BSDS500 dataset as cover images using the respective encryption algorithm (with the plaintext being the next image after the cover image). The second strategy trained a steganalysis model using cover-ciphertext pairs generated by each specific encryption algorithm and examined how detection accuracy varied with increasing training dataset size. The training set for each encryption algorithm consisted of cover-ciphertext pairs generated by encrypting the last 400 images of the BSDS500 dataset as cover images (again, with the plaintext being the next image after the cover image). The test set for each algorithm was the same as that used in the first strategy. In both strategies, SRNet was trained for only 10 epochs with a learning rate of 10^−4^, no validation set was used, and the final parameters were applied to testing. For the first strategy, a detection accuracy close to 0.5 indicates stronger resistance to steganalysis (i.e., random guessing). For the second strategy, a slower increase in detection accuracy as training data grow implies higher steganographic security. Figures 21A and 21B present the corresponding experimental results, respectively.

Based on the results shown in Figure 21A, it can be observed that, when evaluated using the model trained on the public dataset, the detection accuracy of the proposed algorithm is closest to 0.5, indicating performance equivalent to random guessing. This demonstrates that the meaningful ciphertexts generated by the proposed scheme exhibit strong undetectability under generic deep-learning-based steganalysis. Furthermore, as illustrated in Figure 21B, although the detection accuracy increases with the size of the training dataset under the second evaluation strategy, the proposed algorithm exhibits the slowest growth rate among all compared methods. This indicates that substantially more training samples are required for a steganalysis model to achieve comparable detection performance, further confirming the robust resistance of the proposed encryption scheme against deep-learning-based steganalysis.

## Discussion

This paper proposes a novel ND-ICNTC chaotic system that achieves fast sequence generation with a controllable diffusion speed. Comprehensive dynamical analyses demonstrate its strong randomness and suitability for cryptographic applications. Based on this chaotic system, a secure image encryption framework is constructed by integrating compressed sensing and scrambling mechanisms. To further enhance ciphertext imperceptibility, a histogram-reorganizing embedding strategy is introduced. This strategy adaptively reshapes the histogram of the noise-like ciphertext to an optimal distribution under a given transform kernel, thereby minimizing cover distortion during GLSB embedding. Meanwhile, the proposed method breaks the conventional value range of noise-like ciphertexts in image encryption, effectively disturbing statistical characteristics and providing enhanced resistance to statistical analysis attacks. Experimental results show that the proposed method achieves ciphertext PSNRs as high as 51 and 49 dB in the spatial and wavelet domains, respectively, representing improvements of more than 3 and 1.3 dB over existing approaches. In addition, both conventional statistical steganalysis and deep-learning-based steganalysis indicate that the proposed encryption scheme exhibits the strongest resistance against detection. These results demonstrate that the proposed method provides excellent visual quality and imperceptibility, ensuring the secure transmission of visually meaningful ciphertexts.

### Limitations of the study

This study has certain limitations. Specifically, the compressed sensing reconstruction is not analyzed in depth, and further improvements in reconstruction quality are possible compared with deep-learning-based reconstruction approaches. Future work will explore deep compressive sensing and optimized histogram-reorganizing embedding to enhance reconstruction quality and steganalysis resistance, while evaluating robustness under real-world distortions.

## Resource availability

### Lead contact

Requests for further information and resources should be directed to and will be fulfilled by the lead contact, Prof. Jianjun Li (lijjcan@gmail.com).

### Materials availability

This study did not generate new unique reagents.

### Data and code availability

• Data reported in this paper will be shared by the lead contact upon request.
• Code: https://github.com/jsw1995/MCIE_ND-ICNTC_HR.
• Any additional information required to reanalyze the data reported in this paper is available from the lead contact upon request.

## Acknowledgments

This work was supported in part by the 10.13039/501100001809National Natural Science Foundation of China under grants 62471170 and 61871170 and by Zhejiang Province Fund: LQ21F020005.

## Author contributions

Conceptualization, methodology, and writing – original draft, S.J.; validation, investigation, and editing, Y.H. and J.L.; funding acquisition and supervision, J.L.

## Declaration of interests

The authors declare no competing interests.

## STAR★Methods

### Key resources table

**Table:** 

REAGENT or RESOURCE	SOURCE	IDENTIFIER
**Software and algorithms**		

MATLAB R2018a	MathWorks.Inc	https://ww2.mathworks.cn/products/matlab.html
CVG Database	Computer Vision Group University of Granada	https://ccia.ugr.es/cvg/dbimagenes/c512.php
DIV2K Database	N/A	https://data.vision.ee.ethz.ch/cvl/DIV2K/
BSDS100 Database	N/A	https://www2.eecs.berkeley.edu/Research/Projects/CS/vision/bsds/
Code	N/A	https://github.com/jsw1995/MCIE_ND-ICNTC_HR

### Method details

In this study, all experiments were conducted on a personal computer equipped with MATLAB R2018a. The system was configured with an Intel i7 processor, 32 GB of RAM, a 5 TB hard drive, and ran the Windows 11 operating system. Experimental evaluations were performed using three publicly available datasets, namely CVG, DIV2K, and BSDS100. All software tools and datasets used in this work are publicly accessible and are listed in the key resources table.

### Quantification and statistical analysis

#### Image quality analysis

Peak signal-to-noise ratio (PSNR) and structural similarity (SSIM) are employed to evaluate the visual quality of the meaningful ciphertext images. PSNR is defined as

(Equation 40) $$PSNR=10\times \log_{10}\left(\frac{255^{2}\times m\times n}{\sum_{i=1}^{m}\sum_{j=1}^{n}{\left(X\left(i,j\right)-Y\left(i,j\right)\right)}^{2}}\right)$$

where *m* and *n* denote the width and height of the image, respectively, and *X*(*i*, *j*) and *Y*(*i*, *j*) represent the pixel values of the reference image and the test image at position (*i*, *j*). SSIM is defined as

(Equation 41) $$\left\{\begin{array}{c} l\left(X,Y\right)=\frac{2\mu_{X}\mu_{Y}+C_{1}}{\mu_{X}^{2}+\mu_{Y}^{2}+C_{1}}\\ c\left(X,Y\right)=\frac{2\sigma_{X}\sigma_{Y}+C_{2}}{\sigma_{X}^{2}+\sigma_{Y}^{2}+C_{2}}\\ s\left(X,Y\right)=\frac{\sigma_{XY}+C_{3}}{\sigma_{X}\sigma_{Y}+C_{3}}\\ SSIM\left(X,Y\right)=l\left(X,Y\right)\times c\left(X,Y\right)\times s\left(X,Y\right) \end{array}\right)$$

where *μ*_*X*_ and *μ*_*y*_ are the average values of *X* and *Y*, *σ*_*X*_ and *σ*_*Y*_ are variance values of *X* and *Y*, respectively, *σ*_*XY*_ is the covariance of *X* and *Y*,*C*1 = (*k*1*L*)^2^, *C*2 = (*k*2*L*)^2^, *C*3 = *C*2 , *k*1 = 0.01, *k*2 = 0.03, *L* = 255 is the gray level.

#### Differential statistical analysis

Differential attacks aim to reveal potential relationships between plaintext images and their corresponding ciphertexts by introducing slight modifications to the plaintext and analyzing the resulting differences in the encrypted images. In this study, three standard metrics are employed to evaluate the resistance against differential attacks: the number of pixels change rate (NPCR), the unified average changing intensity (UACI), and the structural similarity index (SSIM). NPCR measures the percentage of different pixel values between two ciphertext images generated from slightly different plaintexts and is defined as

(Equation 42) $$NPCR=\frac{1}{m\times n}\overset{m}{\sum }\overset{n}{\sum }|\;\text{Sign}\;\left(X\left(i,j\right)-Y\left(i,j\right)\right)|$$

The function Sign(.) equals 1 when its argument is nonzero and 0 otherwise. The unified average changing intensity (UACI) is defined as:

(Equation 43) $$UACI=\frac{1}{M\times N}\overset{M}{\underset{i=1}{\sum }}\overset{N}{\underset{j=1}{\sum }}\frac{\left|X\left(i,j\right)-Y\left(i,j\right)\right|}{L-1}\times 100\%$$

*L* denotes the number of gray levels in the image (e.g., for an 8-bit image, *L* = 256 and *L* − 1 = 255).

## References

[1] Alawida M. (2023). A novel chaos-based permutation for image encryption. J. King Saud Univ. Comput. Inf. Sci..

[2] Chen Z., Chen Z., Long B., Liu T., Yao M. (2026). A cross-channel color image encryption scheme based on a novel 4d hyperchaotic system and 3-layer peano curve. Expert Syst. Appl..

[3] Wen H., Lin Y., Kang S., Zhang X., Zou K. (2024). Secure image encryption algorithm using chaos-based block permutation and weighted bit planes chain diffusion. iScience.

[4] Lin Y., Xie Z., Chen T., Cheng X., Wen H. (2024). Image privacy protection scheme based on high-quality reconstruction dct compression and nonlinear dynamics. Expert Syst. Appl..

[5] Yan X., Hu Q., Teng L. (2025). A novel color image encryption method based on new three-dimensional chaotic mapping and dna coding. Nonlinear Dyn..

[6] Wang Q., Yang Y., Zhang X. (2026). Real-time medical image encryption algorithm based on punch scrambling and fast hachimoji dna coding. Expert Syst. Appl..

[7] Jiang D., Tsafack N., Boulila W., Ahmad J., Barba-Franco J.J. (2024). Asb-cs: Adaptive sparse basis compressive sensing model and its application to medical image encryption. Expert Syst. Appl..

[8] Guo Z., Chen S.H., Zhou L., Gong L.H. (2024). Optical image encryption and authentication scheme with computational ghost imaging. Appl. Math. Model..

[9] Hu L.Y., Guo Z., Gong L.H. (2026). Color image encryption and authentication algorithm integrating computational ghost imaging with quaternion multi-parameter discrete fractional angular transform. Expert Syst. Appl..

[10] Verma V., Kumar S. (2025). Quantum image encryption algorithm based on 3d-bnm chaotic map. Nonlinear Dyn..

[11] Wang C., Zhang Y. (2022). A novel image encryption algorithm with deep neural network. Signal Process..

[12] Bao L., Zhou Y. (2015). Image encryption: Generating visually meaningful encrypted images. Inf. Sci..

[13] Shiwei J., Jianjun L. (2024). An overview of visually meaningful ciphertext image encryption. Multimed. Tool. Appl..

[14] Yang Y.G., Zhang Y.C., Chen X.B., Zhou Y.H., Shi W.M. (2018). Eliminating the texture features in visually meaningful cipher images. Inf. Sci..

[15] Ye G., Liu M., Yap W.S., Goi B.M. (2023). Reversible image hiding algorithm based on compressive sensing and deep learning. Nonlinear Dyn..

[16] Yang Y., Cheng M., Ding Y., Zhang W. (2023). A visually meaningful image encryption scheme based on lossless compression spiht coding. IEEE Trans. Serv. Comput..

[17] Wu H., Gan Y., Ye G. (2025). Reversible image hiding algorithm based on adaptive embedding mechanism. Expert Syst. Appl..

[18] Yu F.F., Dai J.Y., Liu S.H., Gong L.H. (2023). Visually meaningful quantum color image encryption scheme based on measured alternate quantum walks and quantum logistic mixed linear-nonlinear coupled mapping lattices. Int. J. Theor. Phys..

[19] Yang Y.G., Wang B.P., Yang Y.L., Zhou Y.H., Shi W.M., Liao X. (2021). Visually meaningful image encryption based on universal embedding model. Inf. Sci..

[20] Ye G., Liu S., Xiao X., Hunag X. (2025). Image hiding algorithm based on local binary pattern and compressive sensing. Math. Comput. Simulat..

[21] Yang Y.G., Wang B.P., Pei S.K., Zhou Y.H., Shi W.M., Liao X. (2021). Using m-ary decomposition and virtual bits for visually meaningful image encryption. Inf. Sci..

[22] Li C., Zhang Y., Li H., Zhou Y. (2024). Visual image encryption scheme based on inter-intra-block scrambling and weighted diffusion. Vis. Comput..

[23] Wang X.Y., Wang X.L., Teng L., Jiang D.H., Xian Y. (2023). Lossless embedding: A visually meaningful image encryption algorithm based on hyperchaos and compressive sensing. Chin. Phys. B.

[24] Zhu L., Jiang D., Ni J., Wang X., Rong X., Ahmad M. (2022). A visually secure image encryption scheme using adaptive-thresholding sparsification compression sensing model and newly-designed memristive chaotic map. Inf. Sci..

[25] Chen W., Wang Y., Xiao Y., Hei X. (2023). Explore the potential of deep learning and hyperchaotic map in the meaningful visual image encryption scheme. IET Image Process..

[26] Yang Y.G., Wang B.P., Yang Y.L., Zhou Y.H., Shi W.M., Liao X. (2023). A visually meaningful image encryption algorithm based on adaptive 2d compressive sensing and chaotic system. Multimed. Tool. Appl..

[27] Chen W., Wang Y., Shi C., Sheng G., Li M., Liu Y., Hei X. (2025). Flexible visually secure image encryption with meta-learning compression and chaotic systems. Neural Netw..

[28] Han J., Ye G., Han S. (2026). An image steganography algorithm using selective timestep embedding and diffusion model. Expert Syst. Appl..

[29] Lin Y., Liao Y., Wei Y., Zeng W., Erkan U., Toktas A., Zhang Y., Chen D. (2025). Lightweight image encryption via four-dimensional hénon memristor map and fast block permutation. Nonlinear Science and Control Engineering.

[30] Shiwei J., Jianjun L. (2024). An image encryption algorithm for visually meaningful ciphertext based on adaptive compressed, 2d-iicm hyperchaos and histogram cyclic shift. Multimed. Tool. Appl..

[31] Khurana N., Dua M. (2025). A novel one-dimensional cosine within sine chaotic map and novel permutation–diffusion based medical image encryption. Nonlinear Dyn..

[32] Rehman M.U. (2024). Quantum-enhanced chaotic image encryption: Strengthening digital data security with 1-d sine-based chaotic maps and quantum coding. J. King Saud Univ. Comput. Inf. Sci..

[33] Cao W., Mao Y., Zhou Y. (2020). Designing a 2d infinite collapse map for image encryption. Signal Process..

[34] Feng W., Zhang K., Zhang J., Zhao X., Chen Y., Cai B., Zhu Z., Wen H., Ye C. (2025). Integrating fractional-order hopfield neural network with differentiated encryption: Achieving high-performance privacy protection for medical images. Fractal Fract..

[35] Gao S., Zhang Z., Iu H.H.C., Ding S., Mou J., Erkan U., Toktas A., Li Q., Wang C., Cao Y. (2025). A parallel color image encryption algorithm based on a 2d logistic-rulkov neuron map. IEEE Internet Things J..

[36] Zheng Y., Huang Q., Cai S., Xiong X., Huang L. (2025). Image encryption based on novel hill cipher variant and 2d-igscm hyper-chaotic map. Nonlinear Dyn..

[37] Liao Y., Lin Y., Xing Z., Li Q., Huang G., Chen D., Yuan X. (2025). Using 3d-lmm-based encryption to secure digital images with 3-d s-box and fibonacci q-matrix. IEEE Internet Things J..

[38] Lin Y., Liao Y., Zeng W., Wei Y., Chen D., Yuan X., Li Y., Erkan U., Toktas A., Zhang C. (2026). 3d non-degenerate hyperchaos: Design, analysis, and application in image encryption. IEEE Trans. Consum. Electron..

[39] Jing S., Li J., Tian W. (2023). 2023 IEEE International Conference on Multimedia and Expo (ICME).

[40] Li G., Song X., Xu W. (2025). An n-dimensional discrete attractor with sinusoidal waveform. Nonlinear Dyn..

[41] Wang M., Ding J., Zhang X., Iu H.H.C., Li Z. (2025). A new construction method of n-dimensional discrete sine hyperchaotic map. Nonlinear Dyn..

[42] Dong Y., Zhao G. (2021). A spatiotemporal chaotic system based on pseudo-random coupled map lattices and elementary cellular automata. Chaos Solitons Fractals.

[43] Wang X., Dai X., Wang Y., Wang E. (2025). N-dimensional non-degenerate chaos based on singular value estimation with application in dynamic dna image encryption. Nonlinear Dyn..

[44] Liu X., Tong X., Zhang M., Wang Z. (2024). Constructing of n-dimensional non-degenerate chaotic maps and its application for robust image encryption. Appl. Math. Model..

[45] Cao W., Cai H., Hua Z. (2022). n-dimensional chaotic map with application in secure communication. Chaos Solitons Fractals.

[46] Zhao M., Li L., Yuan Z. (2024). A multi-image encryption scheme based on a new n-dimensional chaotic model and eight-base dna. Chaos Solitons Fractals.

[47] He D., He C., Jiang L.G., Zhu H.W., Hu G.R. (2001). Chaotic characteristics of a one-dimensional iterative map with infinite collapses. IEEE Trans. Circ. Syst. I..

[48] Hénon M. (1976). A two-dimensional mapping with a strange attractor. Commun. Math. Phys..

[49] Li Y., Tang W.K., Chen G. (2005). Generating hyperchaos via state feedback control. Int. J. Bifurcation Chaos.

[50] Refregier P., Javidi B. (1995). Optical image encryption based on input plane and fourier plane random encoding. Opt. Lett..

[51] Chen W., Ji W., Wang Y., Ren J., Sheng G., Hei X. (2025). Visually secure image encryption: Exploring deep learning for enhanced robustness and flexibility. Expert Syst. Appl..

[52] Feng W., Zhang J., Chen Y., Qin Z., Zhang Y., Ahmad M., Woźniak M. (2024). Exploiting robust quadratic polynomial hyperchaotic map and pixel fusion strategy for efficient image encryption. Expert Syst. Appl..

[53] Wen H., Zhang C., Huang L., Ke J., Xiong D. (2021). Security analysis of a color image encryption algorithm using a fractional-order chaos. Entropy.

[54] Wen H., Yu S., Lü J. (2019). Breaking an image encryption algorithm based on dna encoding and spatiotemporal chaos. Entropy.

[55] Wen H., Yu S. (2019). Cryptanalysis of an image encryption cryptosystem based on binary bit planes extraction and multiple chaotic maps. Eur. Phys. J. Plus.

[56] Boehm B. (2014). Stegexpose-a tool for detecting lsb steganography. arXiv.

[57] Boroumand M., Chen M., Fridrich J. (2019). Deep residual network for steganalysis of digital images. IEEE Trans. Inf. Forensics Secur..

[58] Holub V., Fridrich J. (2012). 2012 IEEE International Workshop on Information Forensics and Security (WIFS).

[59] Bas P., Filler T., Pevný T., Filler T., Pevný T., Craver S., Ker A.D. (2011). Information Hiding - 13th International Conference, IH 2011, Prague, Czech Republic, May 18-20, 2011, Revised Selected Papers vol. 6958 of *Lecture Notes in Computer Science*.

